# Brain matters: unveiling the distinct contributions of region, age, and sex to glia diversity and CNS function

**DOI:** 10.1186/s40478-023-01568-z

**Published:** 2023-05-22

**Authors:** Luise A. Seeker, Nadine Bestard-Cuche, Sarah Jäkel, Nina-Lydia Kazakou, Sunniva M. K. Bøstrand, Laura J. Wagstaff, Justyna Cholewa-Waclaw, Alastair M. Kilpatrick, David Van Bruggen, Mukund Kabbe, Fabio Baldivia Pohl, Zahra Moslehi, Neil C. Henderson, Catalina A. Vallejos, Gioele La Manno, Goncalo Castelo-Branco, Anna Williams

**Affiliations:** 1 Centre for Regenerative Medicine, Institute for Regeneration and Repair, Edinburgh Bioquarter, University of Edinburgh, 5 Little France Drive, Edinburgh, EH16 4UU UK; 2 Institute for Stroke and Dementia Research, Klinikum Der Universität München, Ludwig-Maximilians-Universität, Munich, Germany; 3 Munich Cluster for Systems Neurology (SyNergy), Munich, Germany; 4 Laboratory of Molecular Neurobiology, Department of Medical Biochemistry and Biophysics, Karolinska Institutet, 171 77 Stockholm, Sweden; 5 Laboratory of Neurodevelopmental Systems Biology, Brain Mind Institute, School of Life Sciences, École Polytechnique Fédérale de Lausanne (EPFL), 1015 Lausanne, Switzerland; 6 Centre for Inflammation Research, The Queen’s Medical Research Institute, Edinburgh BioQuarter, University of Edinburgh, Edinburgh, UK; 7 MRC Human Genetics Unit, Institute of Genetics and Cancer, Western General Hospital, University of Edinburgh, Edinburgh, EH4 2XU UK; 8 The Alan Turing Institute, 96 Euston Road, London, NW1 2DB UK; 9 Ming Wai Lau Centre for Reparative Medicine, Karolinska Institutet, Stockholm Node, 171 77 Stockholm, Sweden

**Keywords:** Human glia, Developmental origin, Spinal cord, Ageing, Myelin, OPCs

## Abstract

The myelinated white matter tracts of the central nervous system (CNS) are essential for fast transmission of electrical impulses and are often differentially affected in human neurodegenerative diseases across CNS region, age and sex. We hypothesize that this selective vulnerability is underpinned by physiological variation in white matter glia. Using single nucleus RNA sequencing of human post-mortem white matter samples from the brain, cerebellum and spinal cord and subsequent tissue-based validation we found substantial glial heterogeneity with tissue region: we identified region-specific oligodendrocyte precursor cells (OPCs) that retain developmental origin markers into adulthood, distinguishing them from mouse OPCs. Region-specific OPCs give rise to similar oligodendrocyte populations, however spinal cord oligodendrocytes exhibit markers such as *SKAP2* which are associated with increased myelin production and we found a spinal cord selective population particularly equipped for producing long and thick myelin sheaths based on the expression of genes/proteins such as *HCN2*. Spinal cord microglia exhibit a more activated phenotype compared to brain microglia, suggesting that the spinal cord is a more pro-inflammatory environment, a difference that intensifies with age. Astrocyte gene expression correlates strongly with CNS region, however, astrocytes do not show a more activated state with region or age. Across all glia, sex differences are subtle but the consistent increased expression of protein-folding genes in male donors hints at pathways that may contribute to sex differences in disease susceptibility. These findings are essential to consider for understanding selective CNS pathologies and developing tailored therapeutic strategies.

**Supplementary Information:**

The online version contains supplementary material available at 10.1186/s40478-023-01568-z.

## Introduction

The white matter of the human central nervous system (CNS) contains many non-neuronal cells called glia which include oligodendroglia (oligodendrocytes and their oligodendrocyte precursor cells - OPCs), astrocytes and microglia. Glia are of crucial importance for the physiology of the CNS and are also key players in the pathologies of demyelinating, neurodegenerative and neuropsychiatric disorders (reviewed in [5]). Some of these diseases have pathological biases to CNS region, are more common in one sex and increase in incidence with age. Animal model data suggest that functional differences in glia may causally underpin this pathological variation, with particular importance attributed to oligodendroglial involvement in demyelinating diseases, which we focus on here. In mouse, there is morphological and functional diversity in oligodendroglia in different white matter regions [24, 45] linked to differences in tissue environment [79], axon caliber [3], extracellular matrix stiffness [66], and intrinsic factors related to their developmental origin [3, 14]. Regardless of CNS region, there is a functional shift in glia with age; aged rodent OPCs proliferate and differentiate slower [51, 72], aged rodent oligodendrocytes remyelinate white matter demyelinated lesions less efficiently [69], aged rhesus monkeys and humans show more pro-inflammatory activated phagocytic microglia [68, 71] and increasing age is the main risk factor for more severe disability in the demyelinating disease multiple sclerosis [64]. Sex dimorphism in neurodegenerative disease susceptibility suggests that gonosomal genes or hormones may affect CNS function [12, 35]: cultured female neonatal rat OPCs show more proliferation, migration and less differentiation than males [83], and there are more microglia with homeostatic markers in adult female mice [37].

However, despite these data from animal models and their presumed link to disease, white matter glial regional, age and sex diversity is not well understood in humans. Single-nucleus RNA sequencing (snRNAseq) data from post-mortem human samples have been very successfully used to study cellular changes in development [16, 19, 25, 32, 89] or disease [33, 47, 50, 65, 78]. We used this technology to address our hypothesis that in the normal human CNS, there is regional, age and sex-related glial diversity and that if we better understand these differences, we will better understand region, age and sex susceptibilities to CNS diseases.

Here, we characterized human white matter nuclei of healthy post-mortem samples from three different CNS regions: Primary motor cortex (Brodmann area 4; BA4), cerebellum (CB) and cervical spinal cord (CSC), from donors of two different adult age groups (30–45y and 60–75y) and both sexes. We found significant regional heterogeneity with region-specific populations of OPCs and astrocytes, and spinal cord-enriched populations of oligodendrocytes and microglia. Transcriptional variation with age and sex was also identified, with most marked effects in OPCs, microglia and astrocytes. We discuss potential causes and the expected impact of these differences on health and targeting therapies in disease and provide an open-source atlas for researchers as a comparator for pathologies.

## Materials and methods

### Experimental design

**Fig. 1:**
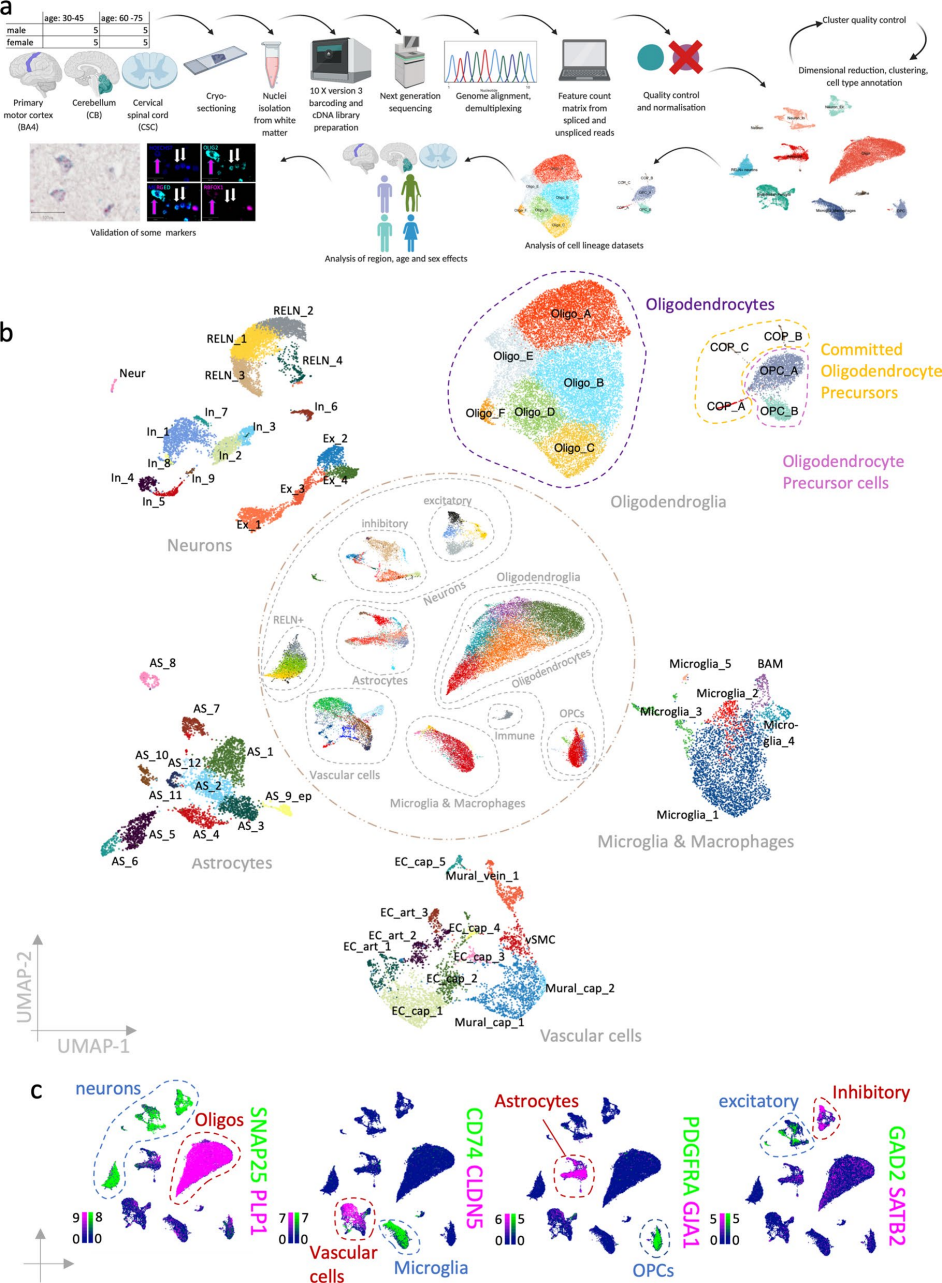
Complete dataset. **a** Schematic of the workflow. **b** UMAP representation of the complete dataset in the centre and clustered cell lineage datasets at the circumference. **c** Feature plots of a selection of canonical cell lineage marker genes in the complete dataset (scale bars show LogNormalized counts). *Oligos* Oligodendrocytes, excitatory = excitatory neurons, inhibitory = inhibitory neurons

For the snRNAseq experiment (Fig. 1A), 20 donors were selected to equally represent two age groups (10 “young adults”: 30–45 years, 10 “old adults”: 60–75 years) and two sex groups (5 male, 5 female within each age group). Each donor donated fresh frozen white matter from the following three tissue regions: primary motor cortex (Brodmann area 4, BA4), arbor vitae cerebelli (CB) and fasciculi cuneatus and gracilis from and cervical spinal cord (CSC) which resulted in a total of 60 samples (Addtional file 1: Table S1). The samples were randomly allocated to 10X chromium chips and then again randomly pooled for cDNA sequencing to reduce batch effects. Validation of bioinformatics results was performed in formalin-fixed paraffin-embedded tissue of the same tissue regions donated by different donors of the same age and sex groups (Addtional file 1: Table S2).

### Human donor tissue

Adult post-mortem unfixed fresh-frozen tissue and formalin-fixed paraffin-embedded tissue were obtained from the MRC Sudden Death Brain Bank in Edinburgh with full ethical approval (16/ES/0084). All samples are verified to have no detectable neuropathology by a consultant neuropathologist (Professor Colin Smith) using published staging/ grading systems, including Braak, NIAA, Thal, Newcastle LBD criteria, LATE pTDP stages, and VCING. Each case has multiple samples assessed, from both hemispheres and multiple cortical, subcortical, cerebellar, brainstem regions plus the cervical spinal cord. Formalin-fixed paraffin-embedded samples were sectioned at 4 µm by the Shared University Research Facilities (SuRF, https://surf.ed.ac.uk/about-us/) (BA4) or by the MRC Sudden Death Brain Bank (CB and CSC).

### Luxol-fast-blue staining with cresyl violet counter stain (LFB-CV)

Fresh frozen tissue was placed in Luxol Fast Blue (LFB) solution (0.1% LFB in 95% ethanol, 0.05% Acetic acid) and incubated at 60 °C overnight. Slides were rinsed with tap water and developed using a saturated Lithium Carbonate solution. Slides were washed and placed in Cresyl Fast Violet (0.1% in 0.07% acetic acid in water) and incubated for 10 minutes at 60 °C. After slides had cooled to room temperature, they were developed using 250 µl Glacial acetic acid in 100% Ethanol. Slides were rinsed, dehydrated in increasing ethanol concentrations (70–100%) followed by incubation in xylene and then they were mounted using Pertex mounting medium. LFB-CV stainings were used to identify white matter (WM) for snRNAseq.

### Bulk RNA extraction and RNA integrity (RIN)—value measurement

RNA was extracted from each fresh-frozen sample by homogenizing multiple sections in 500 µl Trizol (Thermo Fisher Scientific, 15596-026). After 5 min incubation at room temperature 100 µl Chloroform (Vickers Labs LTD, che1570) were added, and samples were mixed by vigorous shaking. Samples were incubated at room temperature for 3 min and then centrifuged at 12,000 g for 15 min. The aqueous phase was mixed with the same volume of 70% ethanol and transferred to silica columns of the Qiagen RNeasy Mini kit (Qiagen, 74104). The manufacturer’s instructions were followed to isolate RNA. For RIN-value measurement 1 µl of RNA was mixed with 5 µl RNA screenTape buffer (Agilent, 5190-6506), incubated at 72 °C for 3 minutes and then loaded into a Tapestation 2200.

### Nuclei isolation

For each sample, 20 tissue cryosections at 20 µm thickness were macro-dissected selecting WM using LFB-CV stainings of adjacent sections (Fig. 1A). For one out of sixty samples it was impossible to dissect white matter without capturing grey matter (cerebellum sample) and therefore it was removed from the experiment. The nuclei PURE isolation kit (Sigma, NUC201-1KT) was used to isolate nuclei: Samples were lysed in 200 µl lysis buffer (PURE lysis buffer containing 1 % 0.1 M DTT, 0.1 % Triton, 2 % SUPERase RNAse inhibitor (Thermo Fischer Scientific AM2694)) on ice and homogenized using syringes and needles of descending needle size (first 27 Gauge then 29 Gauge). 360 µl sucrose cushion (PURE 5M sucrose solution containing 11% PURE Sucrose cushion buffer, 0.11% DTT and 0.02 % RNAse inhibitors) was added to each sample and after mixing, samples were filtered through a 30 µm Sysmex/ Partec CellTrics cell strainer (Wolf Laboratories, 04-004-2326). The filtrate was carefully pipetted on top of 200 µl sucrose buffer. Samples were centrifuged for 45 min at 16,100 g and 4 °C. The supernatant was discarded, and the pellet was washed twice in ice-cold PURE storage buffer containing 0.2 U/ µl RNAse inhibitors. After the second centrifugation at 1000 g for 5 min, the pellet was dissolved in 100 µl PURE storage buffer. Nuclei were stained with Trypan blue (Bio-Rad Laboratories, 1450013) and their concentration was measured using an automated cell counter (Biorad TC20) and standardized to 1,000,000 nuclei per ml. A detailed protocol can be found on protocols.io (10.17504/protocols.io.261genq1og47/v1).

### 10X loading and library preparation

Samples were randomly allocated to 10X Genomics Chromium single cell 3’ chips (Fig. 1A). The 10X Genomics v3 beads, Gem kits and library kits were used according to the manufacturer’s instructions. Library quality was assessed using a Bioanalyzer (Labchip GX Touch 24 nucleic acid analyzer, PerkinElmer, DNA 1K/12K/Hi Sensitivity Assay LabChip with kit DNA high sensitivity reagent kit CLS760672). If libraries failed to have a pronounced peak at the expected fragment size of 450 nm – 500 nm, they were removed from downstream analysis (1 out of 59).

### Next generation sequencing

Nine to ten cDNA libraries were randomly allocated to seven sequencing pools and added at an equimolar concentration of 5 nmol (Fig. 1A). Sequencing was performed by Edinburgh Genomics using a Novaseq 6000 System and a S2 flow cell. Each pool was sequenced on two different lanes to reduce batch effects. We aimed for a sequencing depth of 70,000 reads per nucleus.

### Genome alignment and raw matrix generation

The transcriptome was demultiplexed and aligned with the human reference genome GRCh38 using 10X Genomics Cellranger 3.0.2. Raw data included 33939 genes and 95992 nuclei.

To retrieve feature-count matrices for exonic and intronic reads separately Velocyto (0.17.16) was used with a repeat mask for the human reference genome GRCh38 which was downloaded from https://genome.ucsc.edu/index.html.

### Data quality control

After empty droplets were removed, doublets were detected within each sample using scDblFinder v 1.6.0. After further quality control (see below), 2.2% of nuclei were potential doublets (Additional file 1: Fig. S1) which were retained in the dataset as they marked populations that could be transitional states. We provide doublet information in our metadata and shiny app and discuss their potential effect on analysis results when relevant. Genes with less than 200 copies across both the spliced and unspliced matrices were removed from the analysis. The nucleus quality control was conducted on the spliced and unspliced matrices separately using UMI counts, gene counts and percentage of mitochondrial RNA (only present on spliced matrix). Thresholds for excluding nuclei were first selected as 3 median absolute deviations using the R library Scater [48]. When Scater-set thresholds were too permissive and no nuclei were filtered, manually set thresholds were used. The combined (spliced + unspliced) filtered dataset included nuclei with a minimum of 231 detected genes (mean 1853) and a mean mitochondrial gene percentage of 2.59%. Library sizes did not correlate with post-mortem intervals or RNA integrity values (Additional file 1: Fig. S2a–f), nor did PMI and RIN correlate (Additional file 1: Fig. S2g).

### Normalization

Scran [42] was used to normalize the combined matrix of spliced and unspliced reads for each tissue separately (because they may differ biologically in their amount of gene expression) and to transform data onto a logarithmic scale. Tissue-specific datasets were subsequently merged.

### Dimensional reduction and clustering

Using Seurat [8], the 2000 most variable genes were selected, all genes were scaled and a principal component analysis was performed. The first 25 principal components were used for constructing a Shared Nearest Neighbor (SNN) graph (k = 20) and as input for a Uniform Manifold Approximation and Projection (UMAP). This process was repeated after sample and cluster quality control steps. Louvain clustering was performed at a resolution of 0.8 (0.5 and 1.3 were also tested). The clustering behavior was not influenced by the allocation of a sample to an individual chromium chip or sequencing batch (Additional file 1: Fig. S2h–i). A combination of the following statistics was used to filter for good quality samples: mean UMI count > 490, proportion of spliced vs. unspliced genes < 75%. This resulted in ten samples being removed from downstream analyses (4 BA4, 5 CB, 1 CSC; 6 young, 4 old; 5 male, 5 female). Human donors are known to be genetically diverse and therefore clustering may depend on the individual. Therefore, we considered following criteria to manually curate clusters: Clusters had to contain nuclei of at least 8 donors, while donors were only considered if they contributed at least 2% to the total cluster size.

### Cell type annotation and analysis of subsetted cell lineage datasets

Canonical marker genes were used to annotate cell types as described in the main text and to select oligodendroglia, astrocytes, microglia and macrophages for separate downstream analysis. The 2000 most variable genes were selected, all genes were scaled and a PCA was performed. The first 10 principal components were used for constructing a shared nearest neighbor graph with 20 k- nearest neighbors and as input for a UMAP. Our clustering strategy involved repeated clustering at different resolutions from obvious under- to clear over-clustering and was considered in combination with differential gene expression analyses (see below) and measures of cluster stability at all those resolutions. If functional differences were recognized based on the expression of marker genes, they were considered in the annotation (e.g. excitatory vs. inhibitory neurons). Endothelial cells and pericytes can share the expression of genes that mark blood vessel types. We, therefore, used the scSorter [29] with weighted markers for blood vessel type annotation as we described in Quick et al. [56].

### Differential gene expression analysis

Differential gene expression analyses for the identification of cluster marker genes and genes that are enriched in tissue, age and sex groups were performed using MAST [23] within Seurat (using test. Use = “MAST”) filtering genes for those that had a minimum positive log2-fold change of 0.25 and were expressed by at least 25 % of cells within the cluster/group of interest. All genes that were expressed in less than 60% of nuclei outside the cluster/group of interest were considered for the visual screening of marker genes and as gene ontology input if they had a minimum log2-Fold change of 0.7 and 0.4 respectively and a significant adjusted p-value (<0.05).

### Compositional analysis

We used the R library miloR [15] for differential abundance testing. For buildGraph() 30 nearest neighbors and 30 dimensions were considered and for makeNhoods() the proportion of graph vertices sampled was set to 0.2. When testing for a tissue effect, a pairwise comparison (CSC vs. BA4, CB vs BA4 and CB vs. CSC) was employed with the exemplary additional argument model.contrasts = c("TissueCB - TissueBA4") in the testNhoods() function. Currently, no other fixed effects can be included in the same model when a contrast is investigated. Sex and age had a much smaller effect when tested alone and will not considerably affect tissue variance whereas it is possible that tissue origin may increase or mask age and sex effects. Therefore, we accounted for tissue and the other factor (sex in the case of age and age in the case of sex) in the same model when testing those factors (Additional file 1: Fig. S3).

### Label transfer and integration with other datasets

Seurat’s CCA label transfer and integration was applied to our oligodendroglia dataset and a published human [33] and three mouse datasets [24, 44, 62]. Mouse gene symbols were translated to human gene symbols using the R library biomaRt [17]. For the label transfer and the integration, the first 30 and 20 dimensions respectively were used to specify the neighbor search space. The integrated data was re-scaled and dimensionally reduced using the first 20 principal components as input for a UMAP.

We created a correlation matrix between predicted and current labels by counting each occasion of a co-labelling and normalizing counts for cluster size and largest cluster to bring the results on a scale from zero to one. The R library corrplot [81] was used with the argument is.corr = FALSE to visualize cluster label correlations.

### Trajectory analysis

Dynverse [59] was used for the selection of suitable trajectory inference methods for the complete oligodendroglia dataset and the oligodendroglia dataset divided by tissue. Slingshot [74], PAGA and PAGA tree, Angle and Scorpius were the top ranking methods for our dataset based on Dynverse and they were all tested within the Dynverse framework, setting PDGFRA expressing OPC nuclei as start point for the trajectories. Additionally, slingshot [74] and Monocle [77] were run outside of Dynverse [59] first separately for each tissue (BA4, CB, CSC), then for the tissues combined. In Monocle, the starting point was first not determined, but because we know that in vitro OPCs can generate oligodendrocytes, we set the start point to the OPC clusters. Lastly, we used scVelo [4] within R using velociraptor [58] on spliced and unspliced counts that were subsetted for nuclei that were retained in the final dataset. We superimposed UMAP projections onto the new dataset that included RNA velocity [43] calculations.

### Gene ontology

Cluster profiler [87] was used on all significantly differentially expressed genes (adjusted *p*-value < 0.05) with a log2-Fold change of at least 0.4 (which corresponds to at least − 30% difference) to identify significantly enriched biological processes in clusters, and tissue, sex and age groups. The function compareCluster() was used to compare clusters within each cell lineage using the argument “enrichGO”, the human genome-wide annotation [10] and a named list of cluster labels and their significantly differentially expressed genes as ENTREZID. For more in-depth biological process results, enrichGO() was run for each cluster separately and the 20 most enriched processes were plotted.

### Environment and code availability

Earlier steps of the analysis (genome alignment) and computational intensive analyses (such as Monocle [77] on oligodendroglia of all tissues) were performed on the University of Edinburgh’s Linux compute cluster. For later steps R (version 4.1.1.) and RStudio (version 1.3.1056) were used on a MacBook Pro (64-bit) with macOS Big Sur 11.6.1. More information on R library versions used for individual analyses can be found in the GitHub repository (https://gitfront.io/r/user-1167685/QNF6fMTSbeE6/Luise-Seeker-Human-WM-Glia/).

### Immunofluorescence

Human formalin-fixed paraffin-embedded tissue sections were de-waxed and re-hydrated in 2 x 10 min in Xylene, 2 x 2 min in 100 % ethanol, then 2 min in each 95%, 80% and 70% of ethanol. Antigen retrieval was performed by microwaving the samples for 15 minutes in 0.01 M citrate buffer (pH = 6, Vector Laboratories, H-3300) in Millipore water. Sudan Black Autofluorescence Eliminator Reagent (Merck Millipore, 2160) was added to each slide for 30 s – 1 min which were then washed twice for 5 min in TBS with 0.001% Triton. Slides were incubated in 3 % H_2_O_2_ for 10 min and washed. Serum block (TBS + 0.5% Triton + 10% horse serum) was added for 1 h at room temperature. Antibodies were diluted in serum block and added to slides prior to an overnight incubation at 4*°*C. On the next day, slides were washed in TBS + 0.001% Triton (2 x 5 min) and incubated with an appropriate ImmPress HRP secondary antibody (see below) for 1 h. After washing the slides, OPAL tyramide dye was added at a dilution of 1:500 for 10 minutes. After washing them, the slides were boiled in 0.01 M citrate buffer (pH = 6) for either 2.5 minutes and then left in the hot buffer for 20 minutes or boiled for 15 minutes if same-species primary antibodies were used in a following step. Slides were washed (2 x 5 min) and incubated overnight at 4*°*C with the second primary antibody diluted in serum block. The procedure of the second day was repeated until the required number of antibody reactions was completed. On the last day, samples were counterstained with Hoechst (10 min) after the tyramide reaction, washed (2 x 5 min) and mounted.

We used following antibodies at indicated dilutions SPARC (Abcam, ab225716, RRID:AB_2924283, 1:100), OLIG2 (R&D Systems, AF2418, RRID:AB_2157554, 1:100), RBFOX1 (Abcam, ab243727, RRID:AB_2924285, 1:100), FMN1 (Abcam, ab244482, RRID:AB_2924284, 1:100), OPALIN (Abcam, ab121425, RRID:AB_11127935, 1:100), HCN2 (Alomone Labs, APC-030, RRID:AB_2313726, 1:1000), GPNMB (Abcam, ab227695, RRID:AB_2924286, 1:100), IBA1(ab178846, RRID:AB_2636859, 1:50). Following secondary antibodies were used: ImmPress HRP secondary antibodies: horse anti-rabbit (Vector Laboratories, MP-7401-15), horse anti-goat (Vector Laboratories, MP-7405). Following OPAL Tyramide dyes were used: OPAL 570 (Akoya Biosciences, SKU FP1488001KT), OPAL 650 (Akoya Biosciences, FP1496001KT) and OPAL 480 (SKU FP1500001KT).

### BaseScope duplex

We used a BaseScope duplex detection kit (ACD Bioscience, 323800) on formalin-fixed paraffin-embedded tissue according to the manufacturer’s instruction with following optimized times: Target retrieval for 15 min. protease digestion for 20 min, AMP7 and AMP11 incubation for 1h each. We used following probes: PDGFRA (BA-Hs-PDGFRA-3EJ-C1, H1037431-C1), NELL1 (BA-HsNELL1-3zz-st-C2, 902681-C2) and PAX3 (BA-Hs-PAX3-3zz-st-C2, 902701-C2).

### RNA scope

We used the RNAScope Multiplex Fluorescent Reagent kit v2 (323100) according to the manufacturer’s instructions with 15 min target retrieval and 25 min protease treatment. We used following probes: Hs-EBF1-C2 (583141-C2) and PDGFRA (604481).

### Imaging and analysis of imaging data

A Vetra Polaris slide scanner (Perkin Elmer) was used to image IF and colorimetric BaseScope Duplex stainings at 20x objective. RNAscope slides were imaged using an Opera Phenix Plus High-Content Screening System (Perkin Elmer) with an air objective at 20X. The exact number of samples quantified for each stain is shown in Addtional file 1: Table S2. For human IF and RNAscope stains, four fields of view within WM that measured 500 x 500 µm were used for each sample to quantify stainings in QuPath [2] by setting channel-specific thresholds for a positive detection for each field of view (IF) or constant across all fields of view (RNAscope) or by counting manually (FMN1 IF). RNAscope quantification was performed automatically by using Qupath’s algorithm for cell detection and subcellular detection. BaseScope duplex stainings were quantified by exporting each field of view as separate image, randomize the file names for blinding and then manually counting single and double positive cells where at least three PDGFRA mRNA molecules had to be detected to mark an OPC. Most quantification results are visualised as box and whisker plots with overlayed individual data points using the standard geom_boxplot() settings where the horizontal line visualizes the median, the box the hinges the 25th and 75th percentile and the whiskers the full data range within 1.5 inter-quartile ranges from the hinges. Data points outside the whiskers may be interpreted as outliers. Data analysis methods were chosen to best reflect the data structure of the individual datasets and included linear models and Poisson models (Table 1).

**Table 1:** Statistical analysis methods and results for validation data

Validation	Method	Model	Model output					Other statistical output	Number of samples (4 fields of view each quantified)
SPARC + RBFOX1- OLIG2 + (IF)	Linear Model	Cube root (percentage of SPARC + RBFOX1-OLIG2 +) ~ tissue region + age*sex	Predictor	Est	SE	*t*-value	*p*-value	NA	BA4: 6 CB: 4 CSC: 6
			(Intercept)	1.2	0.3	4.06	< 0.001		
			Tissue: CB	− 0.17	0.36	− 0.47	0.64		
			Tissue: CSC	0.74	0.32	2.34	0.023		
			Age Group: Young	− 0.43	0.46	− 0.931	0.356		
			Sex: Male	− 0.02	0.39	− 0.07	0.94		
			Young: Male	0.35	0.61	0.578	0.565		
HCN2 + SPARC + OLIG2 + (IF)	Wilcoxon Rank Sum test	Proportion of HCN2 + SPARC + oligodendrocytes ~ tissue	NA				W = 18.5	*p*-value = 0.025	BA4: 3 CSC: 2
PAX3 + PDGFRA + (BaseScope duplex)	Poisson Model	Count of double positive cells ~ total count of PDGFRA positive + tissue	(Intercept)	− 1.31	0.34	− 3.82	< 0.001	NA	5 BA4 5 CB 7 CSC
			Total count PDGFRA	0.05	0.02	1.97	0.048		
			Tissue: CB	1.50	0.31	4.87	< 0.001		
			Tissue: CSC	1.65	0.30	5.56	< 0.001		
NELL1 + PDGFRA + (BaseScope duplex)	Linear Model	NELL1 + PDGFRA + ~ tissue region	(Intercept)	0.67	0.16	4.3	< 0.001	NA	3 BA4 3 CSC
			Tissue: CSC	− 0.67	0.22	− 2.97	0.007		
EBF1 + PDGFRA + (RNAscope)	linear model	Proportion EBF1 + PDGFRA + ~ age group + tissue + mean cellular autofluorescence	(Intercept)	0.43	0.2	4.09	< 0.001	NA	CB old: 4 CB young: 4 CSC old: 5 CSC young: 5
			Age Group	0.18	0.05	3.64	< 0.001		
			Tissue (CSC)	− 0.32	0.05	− 7.05	< 0.0001		
			Cellular auto-fluorescence	44.00	26.27	1.68	0.099		

*IF* Immuno-fluorescence, *CB* Cerebellum, *CSC* Cervical spinal cord, Est Estimate, *SE* Standard Error

### Data and materials availability

All data necessary to reproduce the results of the present paper are available at https://cellxgene.cziscience.com/collections/9d63fcf1-5ca0-4006-8d8f-872f3327dbe9. R code (that also specifies R library versions) is available at https://github.com/Anna-Williams/Luise_Seeker_Human_WM_Glia. We used ShinyCell [53] to generate interactive shiny apps that are accessible at https://seeker-science.shinyapps.io/shiny_app_multi/.

## Results

### Description and annotation of the complete dataset

We used white matter from three anatomical CNS sites selected for their pronounced structural differences: BA4, CB and CSC. We analyzed these samples over a cohort of 20 British Caucasian donors (60 samples in total) (Fig. 1a, Additional file 1: Table S1). Donors equally represented both sexes and two different age groups “young adults” (30-45 y, 5 males and 5 females) and “old adults” (60–75 y, 5 males and 5 females). After strict sample, cluster and nucleus quality control (see methods, Additional file 1: Fig. S2), we retained 48 samples and 48,104 nuclei (mean number of genes per nucleus: 1853, mean number of UMIs per nucleus: 5450, mean mitochondrial gene percentage 2.59%). We compared our data quality to three previously published snRNAseq datasets of the human CNS [33, 47, 50] and found that our filtering was more stringent which resulted in significantly better nucleus quality, based on higher gene and unique molecular identifier (UMI) counts and a lower mitochondrial gene percentage at the cost of slightly fewer nuclei per sample (Additional file 1:Fig. S2j–m). Post-mortem interval and RNA integrity values do not predict nuclei quality in our data (Additional file 1: Fig. S2a–f). A first-level cluster analysis and marker inspection revealed all expected major cell types: excitatory neurons (as marked by *SNAP25, SLC17A7*), inhibitory neurons (*SNAP25, GAD1*), REELIN-positive neurons (*SNAP25, RELN*), astrocytes (*GJA1, GFAP*), microglia and macrophages (*CD74, P2RY12*), endothelial cells and pericytes (*CLDN5, NOTCH3*), oligodendrocytes (*PLP1, CNP*), their precursor cells (*PDGFRA, PTPRZ1*) and immune cells (*HLA-A, PTPRC*) (Fig. 1b–c). We subsetted the data for all main cell lineages and re-clustered each resulting dataset allowing finer distinction of cellular populations, identifying 11 oligodendroglia, 11 astrocyte, 6 microglia and macrophage, 11 vascular and 18 neuronal clusters that expressed distinct cluster marker genes (Fig. 1b, Additional file 2).

### Oligodendroglia are transcriptionally heterogeneous

**Fig. 2:**
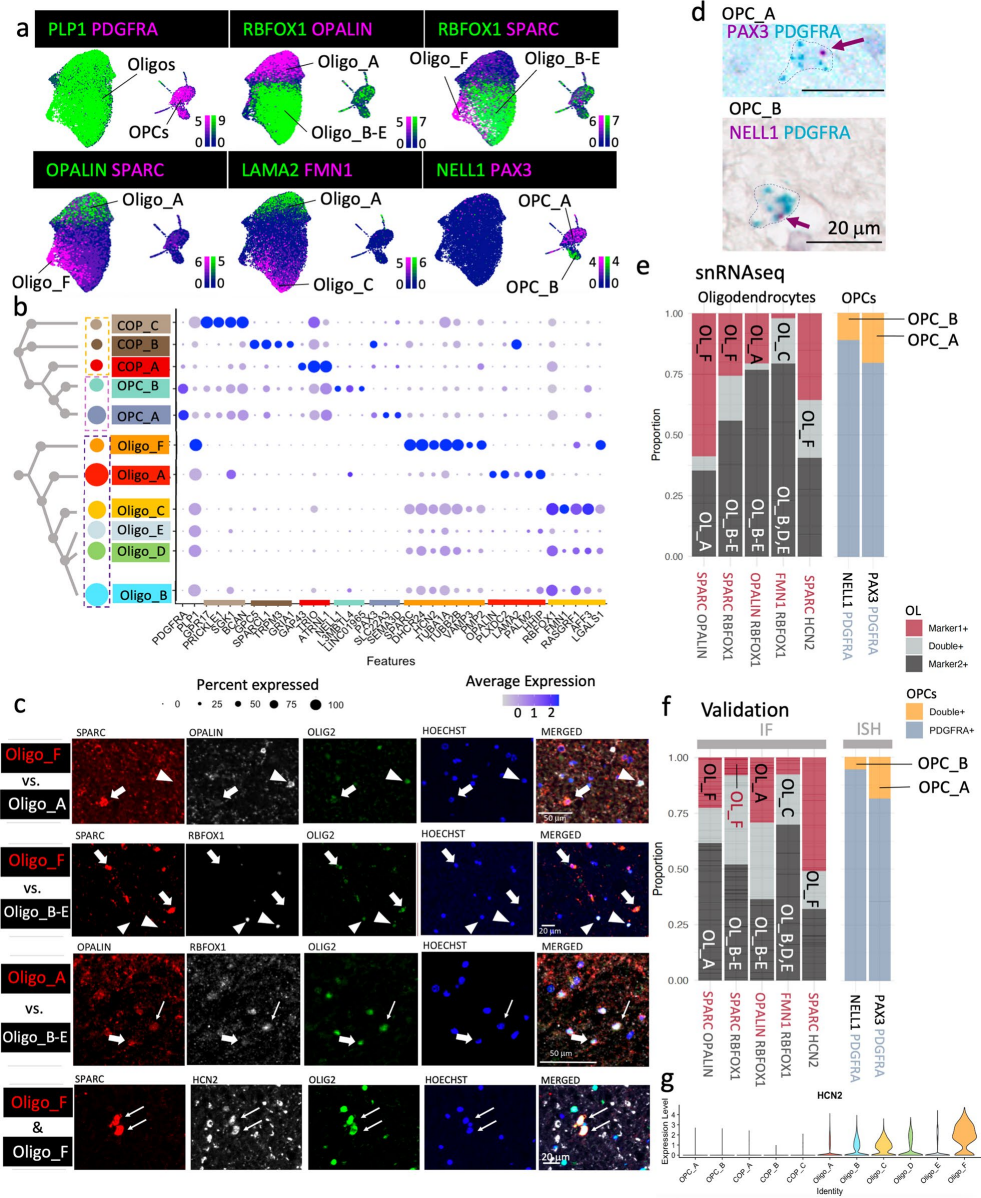
Oligodendroglia cluster segregation and validation. **a** Feature plots of subsetted oligodendroglia that qualitatively show the expression of lineage marker and cluster marker genes (scale bars show LogNormalized counts). *PLP1* marks oligodendrocytes (Oligo) and *PDGFRA* oligodendrocyte precursor cells (OPCs). **b** Dot plot of a selection of marker genes for committed oligodendrocyte precursor cells (COP_A-C), oligodendrocyte precursors (OPC_A & B) and oligodendrocytes (Oligo_A-F) showing cluster segregation. The dendrogram visualizes the relationship of clusters based on 2000 most variable genes. **c** Validation of oligodendrocyte cluster markers by immuno-fluorescence stainings at the protein level. OLIG2-positive oligodendrocytes show either one cluster marker (fat arrow) or a second cluster marker (arrowhead), only with the occasional double-positive cell (thin arrow). SPARC-positive Oligo_F cells also express HCN2. **d** BaseScope duplex stainings for OPC cluster validation. Dotted line delineates the cell boundary with arrow pointing at cluster marker signal. PDGFRA marks all OPCs with PAX3 labelling OPC_A and NELL1, OPC_B. **e, f** Proportion of oligodendroglia expressing cluster markers by pairs in our snRNAseq dataset **e** colour-coded by single markers or overlap and **f** in the validation datasets by immunofluorescence (oligodendrocytes (OL), OLIG2 +) or BaseScope Duplex (OPCs – PDGFRA +), showing good concordance. For example, OL_F (cluster Oligo_F) is SPARC- positive but RBFOX1-negative (second bar in **f**) and as positive for HCN2 and SPARC (5th bar in (f)). *IF* immuno-fluorescence, *ISH* In situ hybridization. **g** Violin plot showing that most HCN2 expression is in the Oligo_F cluster

In our analysis, we focused on oligodendroglia, the most abundant cell lineage in the human white matter. The cluster analysis revealed six oligodendrocyte clusters (positive for myelin genes such as *PLP1, MBP, MAG*), two OPC clusters (positive for *PDGFRA, CSPG4* and *BCAN*) and three committed oligodendrocyte precursor (COP) cell clusters (positive for *GPR17, GPC5* or *GAP43*), confirming that oligodendroglia are heterogeneous in the healthy adult human CNS (Fig. 2). We validated cluster markers (Fig. 2a–b) using immuno-fluorescence stainings for oligodendrocytes (Fig. 2c) and in situ hybridization (Fig. 2d) for OPCs on formalin-fixed paraffin-embedded sections of different donors (Addtional file 1: Table S2) and our quantification shows that the proportion of single and double positive cells for each cluster marker pair is similar to the RNAseq data (Fig. 2e–f).

We found three major populations of oligodendrocytes that are very different in their gene expression: Oligo_A, Oligo_B-E and Oligo_F. They almost mutually exclusively express the markers *OPALIN*, *RBFOX1* and *SPARC* respectively (Fig. 2a–c, e–f), however, Oligo_B-E (*RBFOX1*+) are subclustered based on the expression of additional genes: Oligo_B: *AFF3*+ *LGALS1*- *FMN1*-; Oligo_C: *FMN1*; Oligo_D: *LGALS1*+ *FMN1*- *SPARC*-; Oligo_E: *HHIP*+ *OPALIN*- *AFF3*- (Fig. 2a, b, Additional file 2). Oligo_A and F are *RBFOX1*-negative and strikingly different; Oligo_A expresses *OPALIN, PLXDC2, LAMA2 and PALM2*, and Oligo_F expresses *SPARC, DHCR24, TUBA1A, TUBB2B, PMP2 and HCN2* (Fig. 2b). Gene ontology analysis shows functional differences among oligodendroglia clusters (Additional file 3). For example, for Oligo_A, 52 enriched pathways were identified (Additional file 3) including those important for interaction with neurons, such as axon development, regulation of neuronal projections and GTPase-mediated signal transmission (underpinned by genes such as *STARD13*, *AUTS2* and *ANK3*) and the expression of *ANK3* and *OPALIN* suggests a link with paranodal development and/or maintenance [11, 86] (Additional file 1: Fig. S4, Additional file 3). Oligo_F expresses the novel oligodendrocyte marker *SPARC* and is mostly found in CSC (see below). Gene ontology analysis for Oligo_F markers revealed 405 statistically enriched terms (Additional file 3) including 19 terms based on genes such as *MIF* and *GSTP1* suggesting an immune-related function (Additional file 1: Fig. S5). However, these are different from the immune oligodendroglia we previously described [20], as these oligodendrocytes do not express MHC-2 genes, *CD74* nor *CTSS,* as expected in a healthy cohort. Instead, these immune-related gene ontology terms may reflect the expression of genes related to membrane fusion processes that have been shown to play a role in oligodendrocyte membrane extension, for example by the expression of the vesicle-associated membrane protein 3 (VAMP3) [39] which is indeed more highly expressed in Oligo_F (Fig. 2b). Furthermore, Oligo_F also shows enriched genes related to sterol, cholesterol and myelin production (Additional file 3), and the highest levels of *HCN2,* a gene that is important for the formation of longer myelin sheaths in mice [75] (Fig. 2g). This suggests that Oligo_F is a spinal cord-enriched cluster that contributes to the longer and thicker myelin sheaths of the human spinal cord compared to the brain [3].

### Oligodendroglia clusters correlate with CNS region

**Fig. 3:**
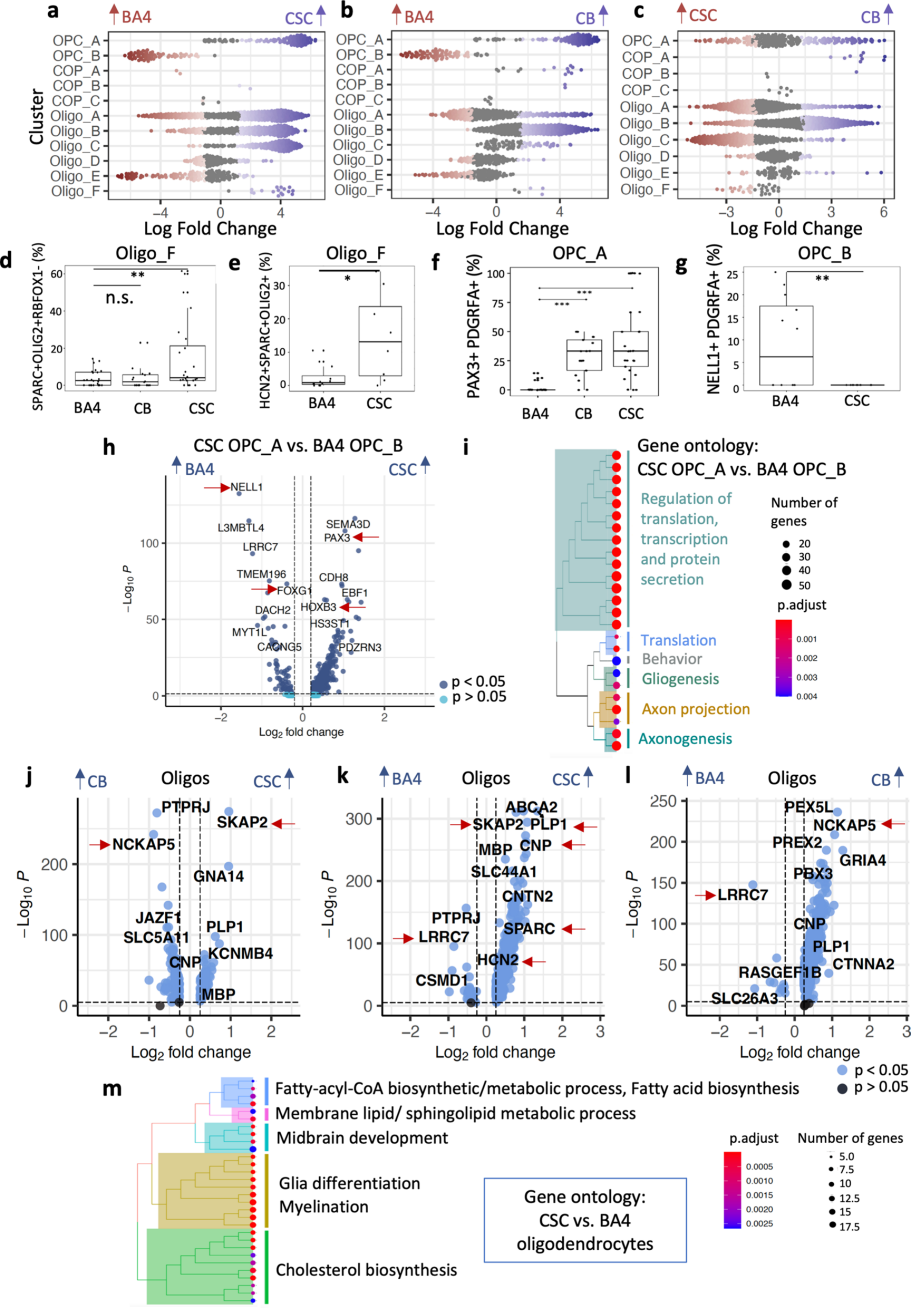
Regional variation in the oligodendroglial population. Differential abundance analysis using Milo shows regional differences between **a** CSC and BA4, **b** CB and BA4 and **c** CB and CSC. Significant increases are coloured in red/blue according to region (FDR < 0.1). Each dot represents a neighbourhood consisting of an average of 50–100 cells. OPC_A is more abundant in CB and CSC, and OPC_B in BA4. Oligo_C and Oligo_F are enriched in the spinal cord compared to the other regions. Oligo_F is a smaller cluster but strikingly different from the other oligodendrocytes in its transcriptome including genes associated with astrocytes (such as SPARC) and Schwann cells (MPZ) while clearly not being an astrocyte or Schwann cell based on the absence of relevant lineage markers. **d, e** Quantification by immunofluorescence (shown in Fig. 2c) showing the increased abundance of Oligo_F in the human spinal cord using the markers **d** SPARC + OLIG2 + RBFOX1- and **e** HCN2 + SPARC + OLIG2 + . **f, g** Quantification by BaseScope duplex of regional selectivity of OPC_A and B (example in Fig. 2d) showing that **f** *PAX3*-positive OPC_A are more abundant in CB and CSC and **g** *NELL1*-positive OPC_B are BA4 specific. Plots visualize percentage of total OLIG2 **d**, **e** or PDGFRA **f**, **g** -positive cells. Boxes in plots visualize median and 25th and 75th percentiles and whiskers mark range up to 1.5 * inter-quartile ranges to show potential outliers. See Table 1 for number of samples, statistical tests used and results and Addtional file 1: Table S2 for sample information. **h** Volcano plot showing differential gene expression between BA4 OPC_B and CSC OPC_A. **i** Gene ontology analysis based on differentially expressed genes of CSC OPC_A in comparison to BA4 OPC_B as shown in h) (complete version in Additional file 1: Fig. S7). Differentially expressed genes in oligodendrocytes (oligos) with tissue region **j–l** with red arrows indicating genes that are discussed in the main text. **m** Gene ontology terms of genes enriched in spinal cord oligodendrocytes in comparison to brain oligodendrocytes. (Complete version in Additional file 1: Fig. S8)

Focussing on Oligo_F, to statistically test whether this cluster is more abundant in CSC, we used the R library Milo [15] which allows for differential abundance testing based on subdividing clusters into neighborhoods and employs an algorithm borrowed from differential gene expression analyses to test if those neighborhoods contain more nuclei of one or the other CNS region. A pairwise comparison of each tissue region revealed that indeed Oligo_F is enriched in CSC (Fig. 3a–c, also shown in Additional file 1: Fig. S6). Validation using two different immuno-fluorescence stainings on formalin-fixed paraffin-embedded samples from different donors, based on the observation that Oligo_F is positive for *SPARC* and *HCN2* but negative for *RBFOX1,* confirmed that Oligo_F is enriched in CSC: both SPARC+RBFOX1- cells (Fig. 3d) and SPARC+ HCN2+ cells (Fig. 3e) are more abundant in CSC compared to the other regions in the validation datasets.

The differential abundance testing also revealed that the two identified OPC clusters are region-specific: OPC_A expresses the Paired Box 3 gene *PAX3* and the cation transporter gene *SLC22A3 (*Fig. 2a–b, d) and is enriched in the posterior CNS-regions (CB and CSC), while OPC_B is more abundant in the anterior BA4. OPC_B is marked by the expression of *NELL1* (Fig. 2a–b, d) which has epidermal growth factor like domains, is predicted to be involved in cell differentiation and has not been previously described in OPCs. We confirmed this OPC segregation using BaseScope duplex by co-labelling *PDGFRA* with either *PAX3* or *NELL1* (Fig. 2d; Fig. 3f–g). In mouse spinal cord, PAX3 is a marker for the third dorsal developmental wave of OPCs and oligodendrocytes derived from PAX3-positive OPCs have been shown to contribute more to remyelination of spinal cord lesions [91] which suggests that (1) human adult OPCs retain a subset of these developmental origin markers and (2) tissue-specific OPCs may vary in function. To explore this further, we performed a differential gene expression analysis between OPCs from the CSC versus BA4 (as the anatomically most distant regions) that revealed 871 differentially expressed genes (Log2FC > 0.25, adjusted *p*-value <0.05, corresponding to at least 19 % upregulation) (Additioanl file 4: Data S3, Additioanl file 5: Data S4). Among those genes, we found that BA4 OPCs upregulate *FOXG1,* which plays a critical role in mouse forebrain development [82], and CSC OPCs express more *HOXB3,* which is important for mouse cervical spinal cord development (Fig. 3h) [38]. This is surprising as human oligodendroglial development is known to be largely similar to rodents [34] and yet here we show that OPC patterning genes that are present at E13.5, but downregulated at P7 in mice [44] are retained in the human into adulthood (Additional file 1: Fig. S9). To address the question if retaining these markers is associated with functional variation, we performed gene ontology analyses on differentially expressed genes between CSC and BA4 OPCs and found that BA4-enriched genes are mainly concerned with ion channel regulator activity (molecular function gene ontology), driven by genes such as *CACNG5*, *FGF12*, *FGF14*, *GRID2*, *KCNAB1*, *KCNIP4*, *KCNQ3*. For CSC OPCs, we found 80 significantly differentially regulated biological processes including glial differentiation, axon development and regulation of transcription, translation and protein secretion (Fig. 3i, Additional file 1: Fig. S7). This indicates that human OPCs that are derived from different origins and/or have been exposed to different tissue environments vary in their function, with implications for the pathogenesis of diseases that affect the brain or spinal cord selectively.

### All oligodendroglia clusters differentially regulate genes based on CNS region

We have shown above that Oligo_F, found more in the spinal cord, expresses key genes for the production of longer and thicker myelin sheaths including myelin genes such as *PLP1, CNP,* and *MAG* and also *HCN2* (known to control mouse myelin sheath length [75]) (Fig. 2b, c, g). Next, we asked if other CSC oligodendrocytes also differ from the other two CNS regions. We performed differential gene expression analyses both pairwise between regions across all oligodendrocyte clusters (Fig. 3j–l) and also cluster-wise and found that oligodendrocytes differentially regulated 13, 170 and 191 genes in BA4, CB and CSC respectively (Log2FC > 0.25, adjusted *p*-value < 0.05). Among the upregulated genes in CSC oligodendrocytes were myelin genes and the Src Kinase-Associated Phosphoprotein 2 gene (*SKAP2)* (Fig. 3j–k) which contributes to thicker myelin sheath formation in mouse spinal cord oligodendrocytes [28]. *SKAP2* is not only expressed by Oligo_F, but also by other spinal cord oligodendrocyte clusters (Additional file 1: Fig. S10), indicating that these all contribute to differences with tissue region. Furthermore, gene ontology analysis performed on differentially expressed genes across all oligodendrocyte clusters confirms that, overall, human spinal cord oligodendrocytes upregulate genes important for cholesterol biosynthesis and myelination (Fig. 3m, Additional file 1: Fig. S8), consistent with mouse [36].

### Age differences in oligodendroglia are largely restricted to OPCs

**Fig. 4:**
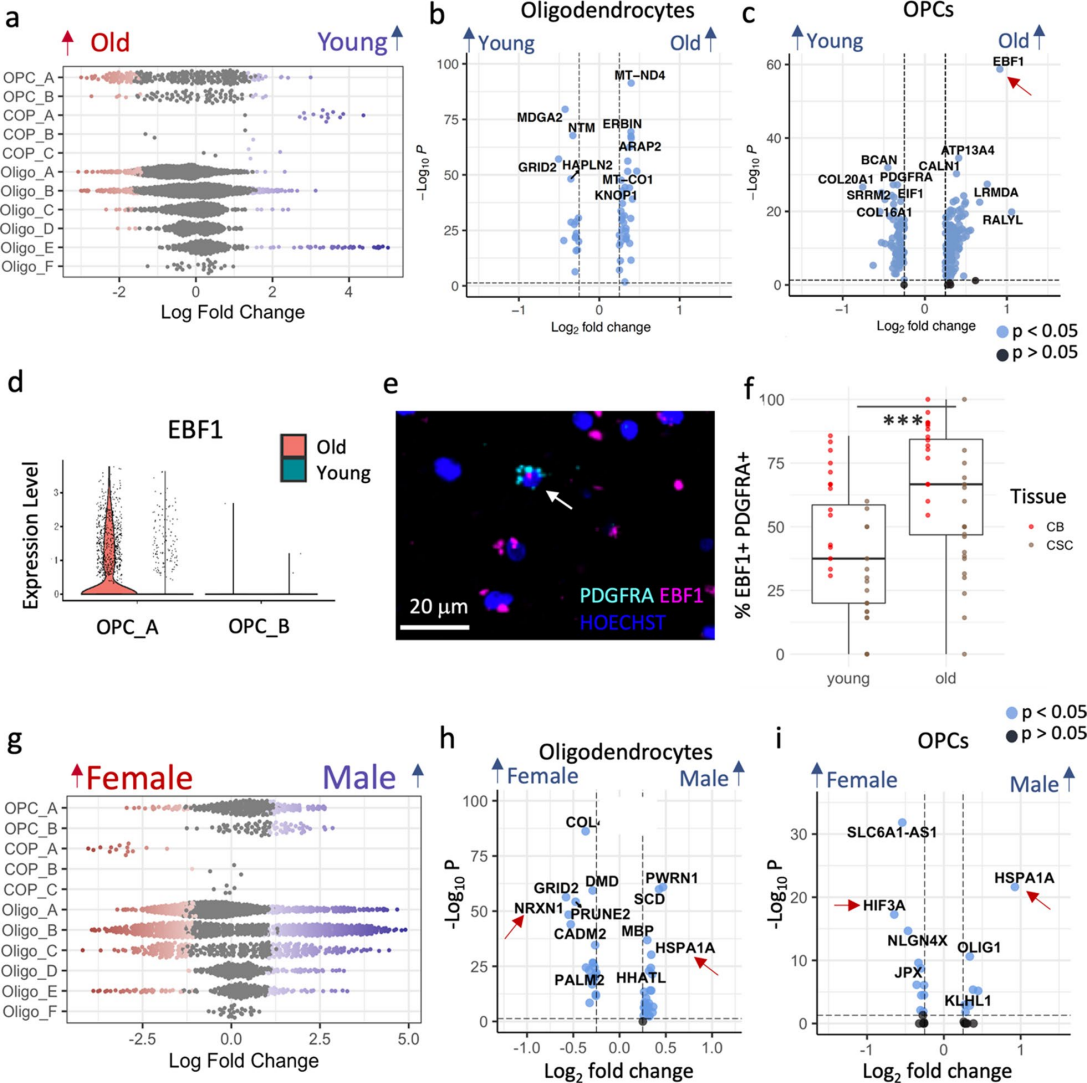
Variation with age and sex in oligodendroglia. **a** Beeswarm plots showing compositional differences with age in oligodendroglia, with red showing significantly more in “old” and blue, significantly more in “young”. Volcano plots showing differentially expressed genes between young and old in oligodendrocytes **b** and OPCs **c**. **d–f** There is increased *EBF1* expression in OPC_A in older donors, shown by violin plot **d**, with RNAscope example of *EBF1* (pink) in *PDGFRA* + OPCs (cyan) with Hoechst (blue) in old donor CSC **e**, and quantification of double-positive EBF1 & PDGFRA cells in validation dataset **f** (Linear model estimate of old age = 0.18, SE = 0.05, *p* < 0,001, N = 18 (see Addtional file 1: Table S2), 4 fields of view each, full model shown in Table 1; boxes visualize median and 25th and 75th percentiles and whiskers mark range up to 1.5 * inter-quartile ranges to mark potential outliers). **g** Compositional sex differences explored using Milo. Differentially expressed genes with sex in oligodendrocytes **h** and OPCs **I** (Gonosomal genes excluded, but shown in Additional file 1: Fig. S11)

As myelin quality deteriorates with age [31] and increasing age is a risk factor for progressive disability in the demyelinating disease multiple sclerosis, we next investigated whether oligodendroglial transcriptional signatures also differ with age in our samples. We first tested for compositional differences using Milo [15] but found that oligodendroglia cluster composition largely did not correlate with donor age except for Oligo_E and COP_A, which show neighborhood enrichment in younger donors (Fig. 4A, Additional file 1: Fig. S3). COPs represent the transition between OPCs and oligodendrocytes during differentiation, needed for myelin maintenance, but produced at a low rate in healthy adult brain. This reduction in COP_A abundance in older donors is consistent with the reduction of capacity of human OPCs to differentiate with age (as seen in rodents [40, 51, 72]) and subsequent reduced myelin integrity [31]. Differential gene expression analyses across the cell lineage-specific dataset, and for each of the oligodendrocyte and OPC cluster separately (Additional file 4, Additional file 5), showed that oligodendrocytes varied little with age, but OPCs showed more age-related transcriptional variation (Fig. 4b–c). Old OPCs expressed less of the early myelin protein gene *PLP1* and less *PDGFRA* (Figure 4c), an OPC membrane receptor mediating proliferation, again consistent with a decline in human OPC function with age. In addition, the spinal cord and cerebellum-specific cluster OPC_A but not OPC_B showed a higher expression of the Early B-cell factor-1 (*EBF1*) in older donors which we validated using RNAScope (Fig. 4c–f). EBF1 is involved in Schwann cell myelination [49] and axonal pathfinding [55] (such proteins are often similarly used for OPC migration) and specific polymorphisms of this gene increase risk of multiple sclerosis [46], suggesting that it may be important to better understand its function in OPCs.

In summary, our results indicate that age-related oligodendrocyte and myelin changes may be influenced by transcriptional changes in the OPC.

### Sex differences in oligodendroglia are subtle

Demyelinating diseases show a variation with biological sex: women are four times more likely to be affected by multiple sclerosis than men, while men show on average a faster progression [35]. Therefore, we next explored sex differences in oligodendroglial transcriptomic signatures. Differential abundance analysis showed few differences (Fig. 4G, Additional file 1: Fig. S3) and differential gene expression analyses across and within clusters show that oligodendroglial gene expression variation with sex is considerably driven by gonosomal genes, such as XIST in female donors and Y-chromosomal genes in male donors: *NLGN4Y*, *TTTY14*, *USP9Y*, *KDM5D*, *ZFY* and *UTY* (Additional file 1: Fig. S11). Although this is not surprising, repression of Y-linked genes in a mouse model of Alzheimer’s disease led to more mitochondrial gene dysfunction and neuroinflammation [9]. Non-gonosomal genes that were differentially regulated between sexes included an upregulation of *NRXN1* in female oligodendrocytes (Fig. 4h), which has previously been linked to Alzheimer’s disease and multiple sclerosis, and of *HIF3A* in female OPCs which is related to oxidative stress (Fig. 4i). Male donors consistently express more heat shock protein *HSPA1A* not only in OPCs and oligodendrocytes, but also in astrocytes and microglia, indicating that male glia may respond differently to stress and vary in their regulation of protein folding mechanisms (Fig. 4h–i, Additional file 1: Figs. S11, S12).

### Adult normal CNS oligodendroglia clusters do not represent transitional states of the differentiation pathway

We were next interested in whether our identified normal adult CNS oligodendrocyte clusters represent transitional states along a differentiation trajectory. This is more likely if (1) OPCs express cell-cycling markers indicating cell-turnover necessary for on-going replacement of differentiating cells, (2) if intermediate cells exist and (3) if similar trajectories can be identified using different methods.

There was no evidence for cycling OPCs (no expression of cell cycling markers e.g. *MKI67* and with Seurat’s cell cycle annotation algorithm [8]) and few detectable intermediate COPs (though more in young donors as above). We used a variety of trajectory inference methods both inside of Dynverse [59] (Angle, Scorpius, PAGA Tree) and standalone (Monocle [77], Slingshot [74], scVelo [4]) but obtained conflicting results as to the overall trajectory and the identity of the most immature oligodendrocyte cluster: Oligo_B (Slingshot, PAGA Tree) or Oligo_A (Monocle, Angle, Scorpius) (Additional file 1: Fig. S13). This is consistent with limited on-going oligodendroglial differentiation in our non-diseased adult human CNS dataset, suggesting either fixed cell end-states or dynamic switching between multiple states which current trajectory inference methods struggle to identify.

### Integrated analysis of oligodendroglia datasets reveals a human-selective oligodendrocyte subtype

**Fig. 5:**
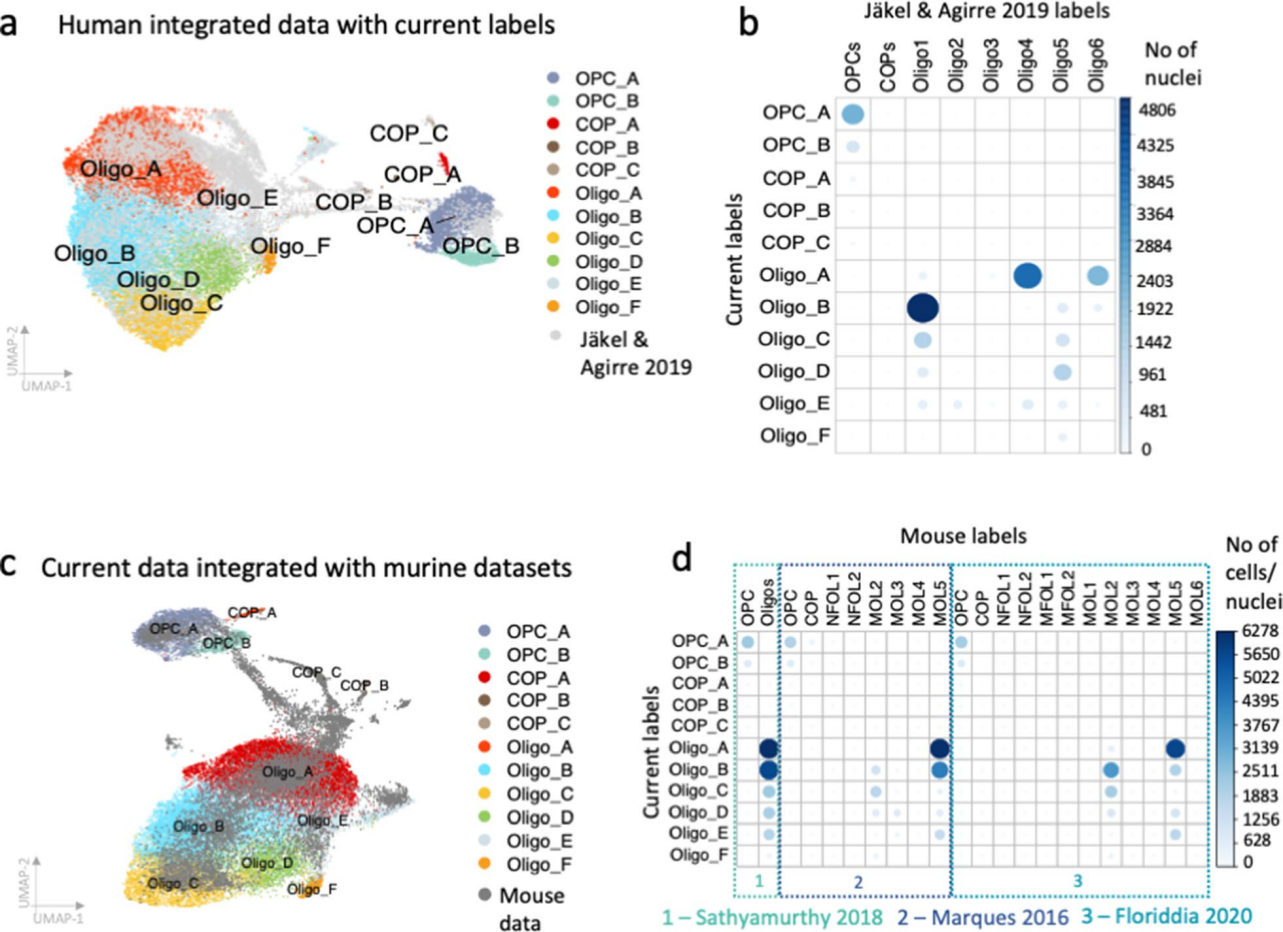
Integration with previously published human and mouse datasets. Current cluster labels include letters to clearly distinguish them from previous cluster labels. **a** Integration with human dataset that includes multiple sclerosis and control brain samples [33]. **b** Correlation of transferred and new labels shows that human datasets overlap well and confirms that oligodendrocyte clusters Oligo2 and 3 are selective for multiple sclerosis whereas Oligo_F is missing in the Jäkel et al. dataset, supporting that Oligo_F is spinal cord-selective. **c–d** Integration with mouse datasets [24, 45, 62] is consistent with the lack of Oligo_F in mouse, even in datasets from spinal cord

To determine how our 11 oligodendroglial clusters relate to published datasets, we transferred the labels of our previous study by Jäkel & Agirre et al [33] which included human samples of subcortical white matter of donors affected by multiple sclerosis and of disease-free controls onto our current dataset and then integrated both datasets (Fig. 5A). The predicted labels fit well with our cluster labels, particularly for cluster Oligo_B which mostly corresponds to cluster Oligo1 and Oligo_A which corresponds best to Oligo6 and Oligo4. Clusters Oligo2 and Oligo3 that are enriched in multiple sclerosis [33] are very rare in our current dataset, confirming that those clusters are multiple sclerosis-selective (Fig. 5b). OPCs in Jäkel & Agirre et al. [33] are of mixed brain regions and correlate to both of our OPC clusters (Fig. 5b). Oligo_F does not map well to any cluster, underlining its spinal cord selectivity (Fig. 5b).

The mouse oligodendrocyte MOL2 expressing *Klk6* has been shown to be enriched in specific regions of the spinal cord [24, 88]. To test whether there is an equivalent of Oligo_F in mouse spinal cord, we also integrated our oligodendroglia dataset with three murine datasets by Marques et al. [45] (different CNS regions of juvenile mice), Sathyarmurthy et al. [62] (adult mouse lumbar spinal cord) and Floriddia et al. [24] (adult corpus callosum and spinal cord) (Fig. 5c–d). Human and mouse data integrated well, with OPC_A, Oligo_A and Oligo_B correlating particularly well to mouse oligodendroglia. However, intermediate cells between the state of OPCs and oligodendrocytes and newly formed oligodendrocytes that are abundant in the mouse data, particularly in the juvenile data (Fig. 5c–d) are rare the human dataset, again suggesting that human adult CNS cells are not actively differentiating in the absence of disease. The *SPARC*-positive, *RBFOX1*-negative human cluster Oligo_F is missing in the mouse datasets, even in the adult mouse spinal cord samples, suggesting it is human-selective (Fig. 5d). There are known proteomic differences between human and murine CNS myelin including the human-specific expression of Peripheral Myelin Protein 2 (*PMP2*) [26], which is highly expressed in our Oligo_F cluster (without expression of other Schwann cell markers; Additional file 1: Fig. S14) and therefore this may contribute to the observed myelin proteomic species difference.

### Region, age and sex variation in astrocytes

**Fig. 6:**
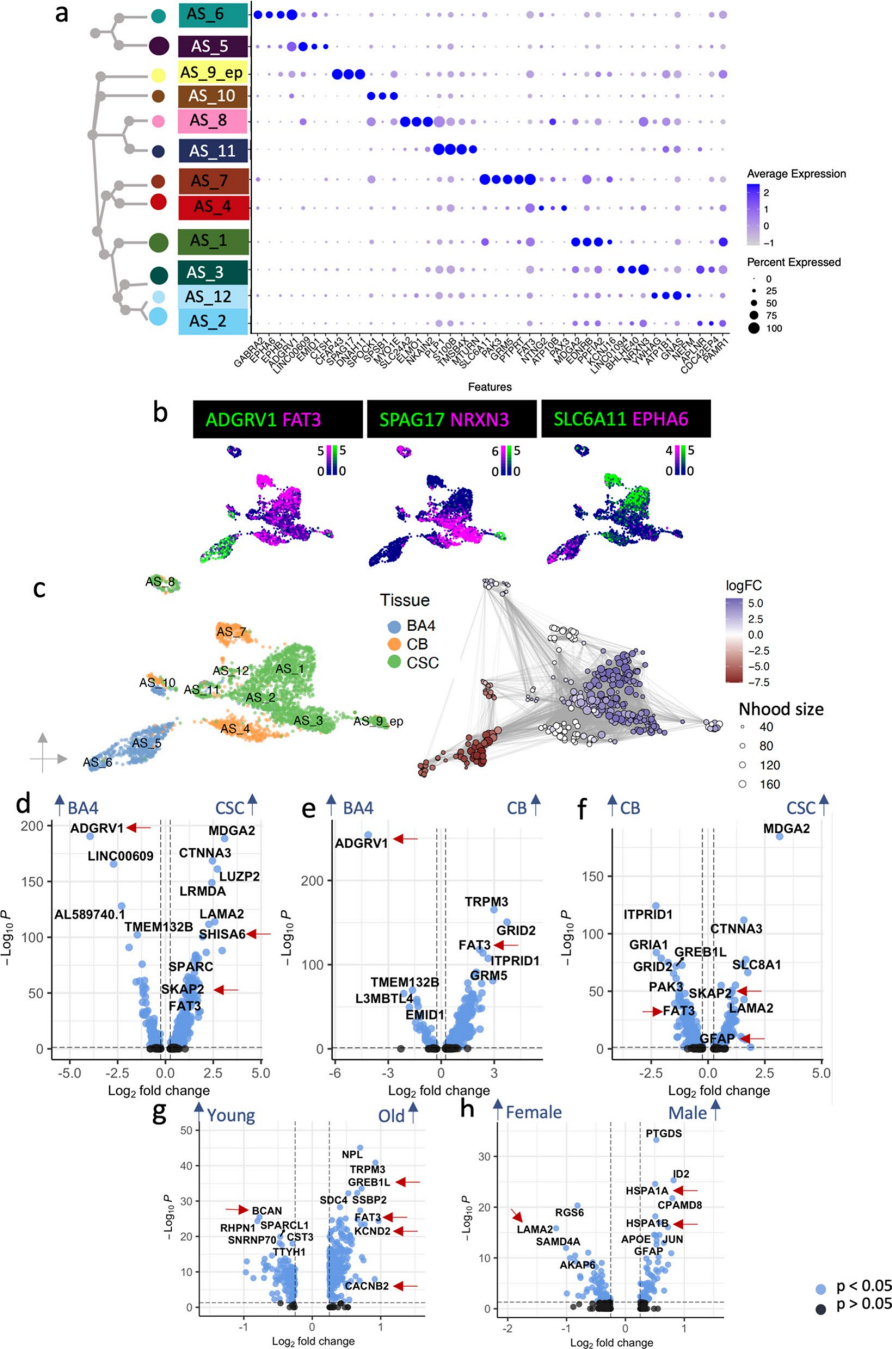
Astrocyte heterogeneity. **a**–**b** Dot plot **a** and feature plots **b** showing astrocyte cluster separation by different markers. (Scale bars show LogNormalized counts) **c** Astrocytes vary considerably with CNS region with enrichment of Milo neighborhoods in CSC in blue, depletion in CSC (and enrichment in BA4) in red, and white are CB selective. Pairwise DGE analysis across all astrocyte clusters shows variation in gene expression between **d** CSC vs. BA4, **e** CB vs. BA4 and **f** CSC vs. CB, **g** old and young and **h** male and female donors. (Gonosomal genes excluded, but shown in Additional file 1: Fig. S11). Red arrows indicate genes discussed further in the main text

White matter oligodendroglia are in constant interaction with neighboring glia and so we also analyzed astrocytes and microglia in the same samples. Human grey matter astrocytes are known to be transcriptionally heterogeneous [70, 90], but data for human white matter astrocytes is much sparser [33, 65]. Our analyses identified twelve white matter astrocyte clusters (AS_1 – AS_12) with AS_9_ep representing transcriptionally closely related ependymal cells, all with distinct marker gene expression (Fig. 1b, 6a–b, Additional file 2). Gene ontology analysis suggests that astrocyte functional roles are distinct, with two types (AS_1 and 2) expressing genes concerned with neuronal interactions, axon guidance and synapse organization, three (AS_4 , 6 and 12) related to synapses/synaptic vesicles, AS_5 relate to extracellular matrix organization, AS_7 to the blood brain barrier and AS_10 to cellular motility, signaling and histone modification. Ependymal cells (AS_9_ep) express motile cilia genes (*CFAP43, SPAG17, DNAH6*) in keeping with their function in promoting cerebrospinal fluid flow [73]. Two populations (AS_8 and 11) also express myelin genes. AS_8 contains some identified potential doublets (Additional file 1: Fig. S1), but these are not present in AS_11 and this has previously been described in human astrocytes [67], (Additional file 1: Fig. S15, Additional file 2).

Astrocytes also showed strong heterogeneity with white matter tissue region: Clusters AS_5 and 6 were specific for BA4, clusters AS_4 and 7 were specific for CB and clusters AS_1, 2, 3, 9_ep, 11 and 12 were more abundant in CSC (Fig. 6c), and differential gene expression analyses between regions reflected differences in these cluster marker genes such as increased Adhesion G Protein-Coupled Receptor V1 (*ADGRV1*) in the BA4-specific cluster AS_5 (Fig. 6d, Additional file 4). In addition, CSC astrocytes expressed more *SKAP2*, similarly to oligodendroglia and more Glial Fibrillary Acidic Protein (*GFAP*) as previously reported in mouse [85] (Fig. 6d, f, Additional file 4). This astrocyte regional specificity may help us understand the regional variation in human astrocytopathies [7]. For example, *FAT3* gene mutations are associated with Spinocerebellar Ataxia (SCA), and is more highly expressed in CB astrocytes particularly of older donors (Fig. 6e–g, Additional file 4). Furthermore, CSC astrocytes express more genes associated with synapse formation and gliogenesis (Additional file 1: Fig. S16a) whereas BA4 astrocytes express more genes associated with the extracellular matrix (Additional file 1: Fig. S16b).

Astrocytes change morphology and function upon injury or disease, previously categorized into a neurotoxic A1 type and a neuroprotective A2 type in mice [41], but now considered more of a spectrum [13], but we found no evidence of genes associated with astrocyte activation in our normal adult dataset, not even with increased age (Additional file 1: Fig. S17). However, we did identify differentially expressed genes with older age in astrocytes which include *CACNB2* and *GREB1L*, important for calcium signaling and the potassium voltage-gated channel protein gene *KCND2* (Fig. 6G, Additional file 4). Astrocytes of younger donors express more BCAN, which is important for experience-dependent neuroplasticity and normal cognitive function [21] (Fig. 6g). Sex differences in the astrocyte population beyond the expression of gonosomal genes (discussed for oligodendroglia above) included more *LAMA2* in female astrocytes, related to the blood brain barrier, and more *CPAMD8* (associated with late-onset Alzheimer’s disease [22]) and heat shock protein gene (*HSPA1A, HSPA1B*) expression in male astrocytes (Fig. 6h, Additional file 1: Fig. S11).

### Region, age and sex variation in microglia

**Fig. 7:**
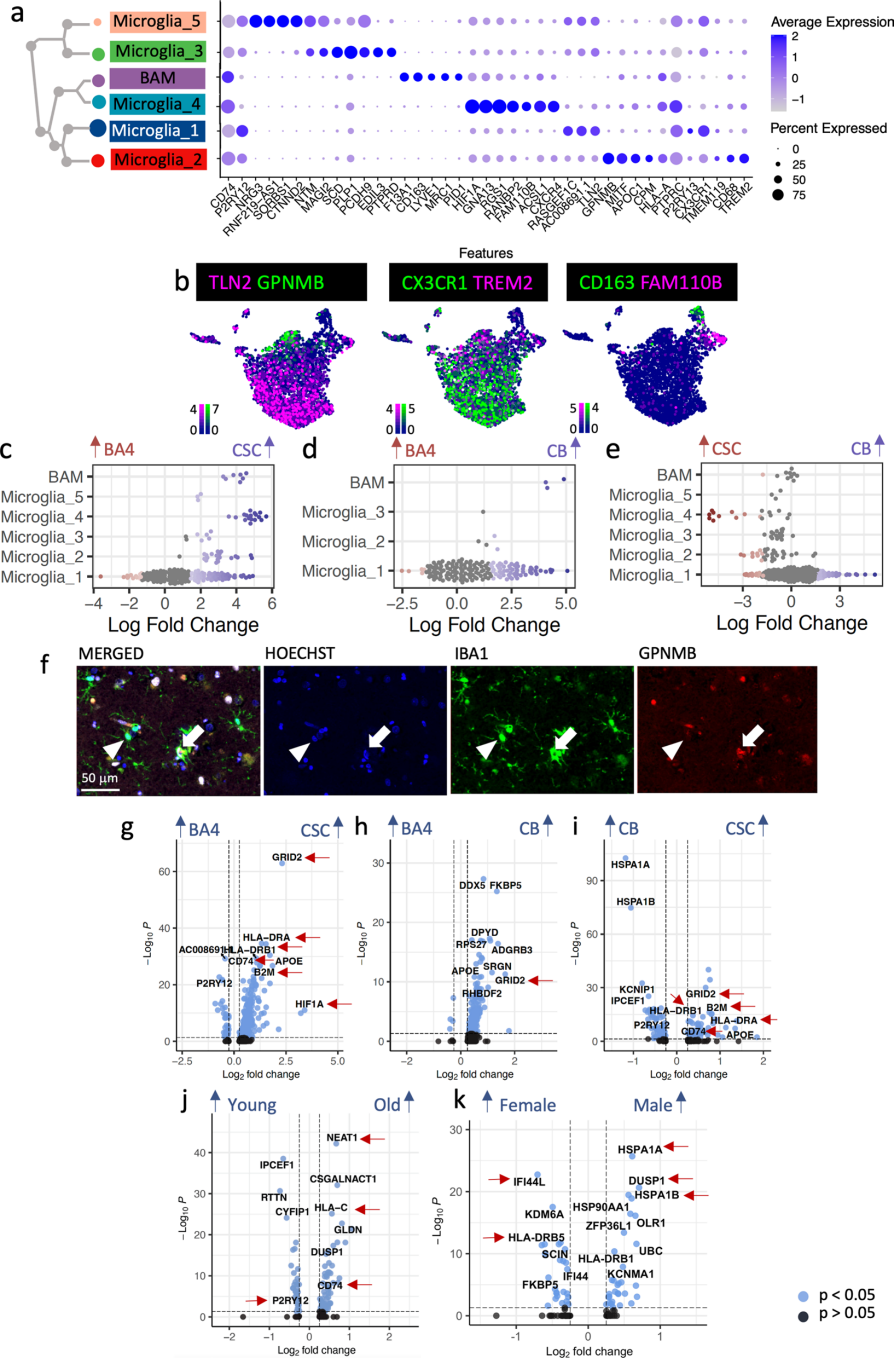
Microglia heterogeneity. **a**, **b** Dot plot **a** and feature plots **b** showing microglial cluster separation by different markers. (Scale bars show LogNormalized counts). **c–e** Microglia vary with CNS region using Milo [15]. **f** Immuno-fluorescence validates that a subset of IBA1-positive microglia expresses the Microglia_2 cluster marker GPNMB (full arrow marks positive cell, arrow head marks negative cell). Pairwise differential gene expression analysis across all microglia clusters shows considerable variation in gene expression between **g** CSC vs. BA4, **h** CB vs. BA4 and **i** CSC vs. CB, **j** old and young and **k** male and female donors. (Gonosomal genes excluded, but shown in Additional file 1: Fig. S11). Red arrows indicate genes discussed further in the main text

We identified five microglia clusters (Microglia_1 – Microglia _5) and one cluster of border-associated macrophages (BAM – positive for *CD163, LYVE1, MRC1*) which express distinct marker genes (Fig. 1b, 7a–b,), but no cells with the phenotype of infiltrating monocyte-derived macrophages, expected to be absent in healthy tissue. Similarly to astrocytes, microglia can shift from a homeostatic to an activated phenotype, characterized by proliferation, chemoattractant-mediated migration, changes in cellular morphology and phagocytosis of damaged cells [30]. Microglia_1 (*RASGEF1C, AC008691.1, TLN2*) express markers for a homeostatic phenotype (*P2RY13* and *CX3CR1*) (Fig. 7a, Additional file 1: Fig. S18, Additional file 2, Additional file 3). Conversely, Microglia_2 (GPNMB) and Microglia_4 (*ACSL1*, *CXCR4*) appear more activated, expressing the phagocytotic markers *CD68* and *TREM2* (Fig. 7a) and with significant leukocyte activation and degranulation gene ontology terms, in spite of being derived from healthy tissue (Additional file 3). However, even in normal adult tissue, activated microglia are needed for regulation of neuronal survival in development, and pruning of synapses, important for circuit refinement [57]. Microglia_3 and 5 express genes classical for neurons or glia such as *NTM, MAGI2, SCD, PLP1*, *NRG3*. Some of these are identified as potential doublets (Additional file 1: Fig. S1) but they may also suggest microglial engulfment of oligodendrocytes and/or synapses (Fig. 7a, Additional file 2, Additional file 3).

The homeostatic Microglia_1 cluster was found similarly in all three tissues but activated Microglia_2 and Microglia_4 were mostly found in CSC (Fig. 7c–e). Microglia_2 expresses *GPNMB* which labels lipid-associated microglia in mice [1], is expressed in microglia in Alzheimer’s disease [63] and which we detected in a subset of microglia using immuno-fluorescence (Fig. 7f). CSC microglia express more major histocompatibility genes and more *CD74* than in other regions, particularly BA4 (Fig. 7g–i), and these findings may be as the blood spinal cord barrier is more permeable than the blood brain barrier, resulting in increased microglial responses to blood-borne stressors. CSC and CB microglia also express more *GRID2* (Fig. 7g–i)*,* previously detected in brain microglia of Alzheimer’s disease donors [27], and more *HIF1A* and *NEAT1,* which may indicate a hypoxic stress response (Additional file 3, Additional file 4, Additional file 5). Border associated macrophages (BAM) were mostly captured for CSC (Fig. 7c–e), probably as meninges, including the perivascular space in which BAMs reside, were captured for this region. (Gene ontology terms for regional differences and activation/homeostatic markers are shown in Additional file 1: Fig. S19 & S20.)

Previous literature suggests that microglia express more activation genes with increased age [60, 68], and our data confirm this; *CD74, NEAT1* and *HLA-C* are more highly expressed in old microglia (Fig. 7J) and markers of homeostasis such *CX3CR1* and *P2RY12* decline with age (Fig. 7j, Additional file 4). However, the relatively subtle difference seen here may be bigger in vivo as snRNAseq is thought to underestimate the expression levels of microglia activation genes in comparison to scRNAseq [76].

Sex differences in the microglia population include more pro-inflammatory genes such as *HLA-DRB5* and *IFI44L* in female microglia (Fig. 7k, Additional file 4), and higher expression of *DUSP1* which modulates microglia towards a homeostatic phenotype [80] in male microglia (Fig. 7k, Additional file 4). Like the other glia, male microglia also express more heat shock protein genes such as *HSPA1A*. These changes suggest sex differences in the link between inflammation and ageing, and may explain some sex dimorphism in neurodegenerative disease susceptibility.

In summary, we show here that in normal adult human WM glia, there is striking variation in transcriptional signatures of all broad cell groups with region, finding both region-specific and region-selective cell populations, with much less variation with age and sex.

## Discussion

We predicted that there would be diversity in normal human glial transcriptional signatures as a read-out of functional variation across CNS regions, age groups and between sexes, to explain the influence of these factors on susceptibility to neurological diseases. We found that transcriptional diversity with CNS region was greater than with age and sex across all glial types, and the striking regional differences between the spinal cord and the brain indicate that we can no longer assume their parity in physiological, pathological or therapeutic response.

Within the oligodendroglia, we found a brain (BA4)-specific and a spinal cord and cerebellum-specific OPC cluster, which retain expression of different developmental markers – unlike in the mouse, where, for example *Hox* genes that label spinal cord OPCs and *Foxg1* that labels brain OPCs are repressed with age [44]. The continued expression of developmental transcriptional factors in our human data indicates that they are needed for adulthood function, differently from mouse. Spinal cord OPCs give rise to similar oligodendrocytes to brain OPCs but have the added capability to differentiate into Oligo_F oligodendrocytes which are selective for the CSC and appear absent in mice. These oligodendrocytes express markers such as *HCN2* that leads to the production of longer and thicker myelin sheaths [75] and high levels of *PMP2* but no other Schwann cell markers. Del Rio Hortega described a Schwannoid and spinal cord-specific oligodendrocyte type (Oligo IV) in the cat CNS (reviewed in [54]), which matches this Oligo_F transcriptional signature. In multiple sclerosis, remyelination of spinal cord lesions is less successful than brain lesions [6] but it is unclear why. We speculate that the functional differences of spinal cord OPCs may alter their ability to differentiate into remyelinating oligodendrocytes and/or that Oligo_F oligodendrocytes, and spinal cord oligodendrocytes generally may be more difficult to replace because of their longer and thicker myelin sheaths. Analysis of snRNAseq data from spinal cord white matter in donors with multiple sclerosis [78] and improving functional in vitro assays for adult human oligodendroglia may shed further light onto their different functions in health and disease.

Other CSC glial differences may relate to the more ‘open’ blood spinal cord barrier compared to the blood brain barrier, therefore likely exposing CSC cells to more blood-derived inflammatory cells/factors explaining activation markers in CSC microglia and alterations in CSC astrocytes, of relevance to pathologies which predominantly affect the spinal cord and for better targeting therapeutics.

In our dataset, age differences were more subtle and we hypothesize that a bigger age disparity, by including a child/adolescent group, would show larger effects. However, *EBF1* has emerged as a marker of aged adult human OPCs, which would be predicted from rodent work [52] to be poorer at initiating remyelination than young OPCs, and this may help us dissect these differences in humans. Changes with age or sex that affect lowly expressed genes will be hard to detect by snRNA-seq [76], and so cell sorting strategies on larger cohorts based on markers identified in the present study with subsequent bulk RNAseq may help highlight such differences. Other physiologically relevant age and sex-related differences may also be post-transcriptional, metabolic or only detectable in response to certain stressors/diseases. Finally, determining whether the subtypes we discovered are associated with local microenvironment or specific cell-cell interactions will require more multiplexed spatially-resolved techniques.

Our results show that human post-mortem tissue is an invaluable source of information highlighting human-specificity of some cellular phenotypes which are difficult to otherwise identify due to the current lack of faithful adult human in vitro models. However, by definition, human post-mortem tissue is limited as it only presents a single snapshot of transcriptional activity without a time course. We attempted to infer a ‘pseudotime’ trajectory between oligodendroglial clusters, but the lack of OPC proliferation and sparsity of intermediate COP stages suggest little oligodendroglia turnover in adulthood in the absence of disease, in agreement with previous radioisotope studies [84], and different from our previous multiple sclerosis dataset [33] with more intermediate cells and disease-specific signatures. Understanding these transitions and how they change in disease will only be solved by use of reporter genes for identified clusters in improved human cell models.

Our findings of marked regional cellular and gene expression differences in normal human post-mortem white matter are critical to consider in strategies for more effective therapeutics for region-specific/selective diseases. For example, our results predict that we will require different pro-remyelinating therapies for successful treatment of spinal cord multiple sclerosis demyelinated lesions compared to brain. For success in translating therapies, we need to embed the consideration of cellular regional, sex and age effects in health and disease throughout our pipeline from preclinical screens to clinical trials - a ‘precision medicine’ approach. We provide these data as an open resource for others for appropriate comparisons with future diseased human CNS cohorts in line with the Human Cell Atlas Project, and in a Shiny app for easy browsing https://seeker-science.shinyapps.io/shiny_app_multi/.

## Supplementary Information

**Additional file 1:** Supplementary Figures and Tables.

**Additional file 2:** Table of cluster marker genes for all cell lineages.

**Additional file 3:** Table of cluster gene ontology terms for all cell lineages.

**Additional file 4:** Table of differentially expressed genes with region, age and sex.

**Additional file 5:** Table of cluster-wise differentially expressed genes with region, age and sex.

**Additional file 6:** Table of gene ontology terms with CNS region, age and sex.

## Data Availability

All data necessary to reproduce the results of the present paper are available at https://cellxgene.cziscience.com/collections/9d63fcf1-5ca0-4006-8d8f-872f3327dbe9. R code (that also specifies R library versions) is available at https://github.com/Anna-Williams/Luise_Seeker_Human_WM_Glia. We used ShinyCell [53] to generate interactive shiny apps that are accessible at https://seeker-science.shinyapps.io/shiny_app_multi/.
