# Supplementary material for: Brain matters: unveiling the distinct contributions of region, age, and sex to glia diversity and CNS function
Source: Acta Neuropathol Commun. 2023 May 22;11:84. doi: 10.1186/s40478-023-01568-z (PMC10204264; doi:10.1186/s40478-023-01568-z)
Supplement: Supplementary file 1 — Additional file 1: Supplementary Figures and Tables. [file 40478_2023_1568_MOESM1_ESM.docx]

Supplementary Materials for

**Brain matters: Unveiling the Distinct Contributions of Region, Age, and Sex to Glia Diversity and CNS Function**

Luise A. Seeker *et al.*

*Corresponding author. Email: [anna.williams@ed.ac.uk](mailto:anna.williams@ed.ac.uk)

**This PDF file includes:**

Figs. S1 to S20

Tables S1 to S2

**Other Supplementary Materials for this manuscript include the following:**

Data S1 to S5


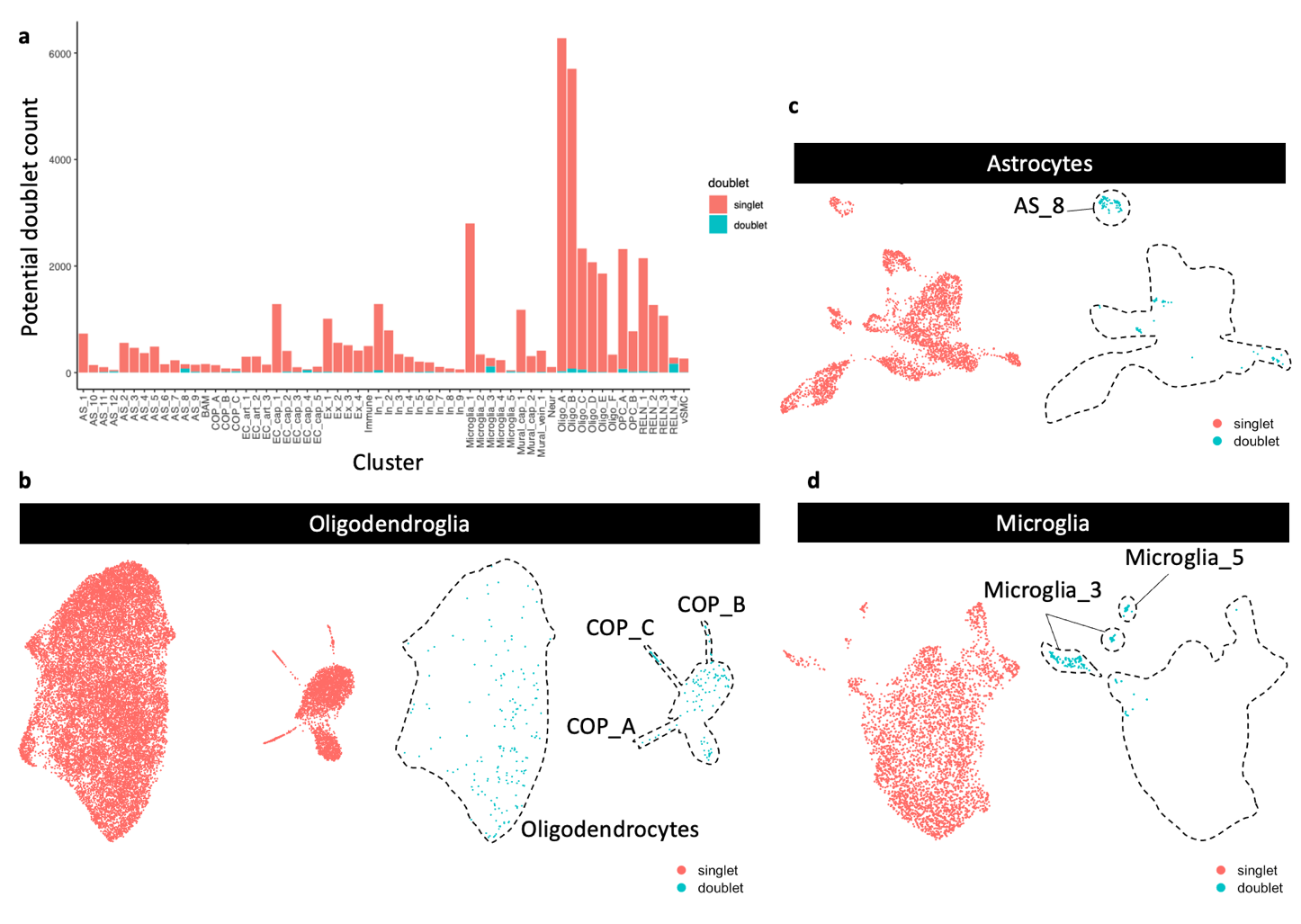


Fig. S1 Doublet detection. Potential doublets were identified using scDblFinder and make up 2.2% of the total dataset and are concentrated in clusters that are accordingly addressed in the main text (AS_8, Microglia_3 and Microglia_5).


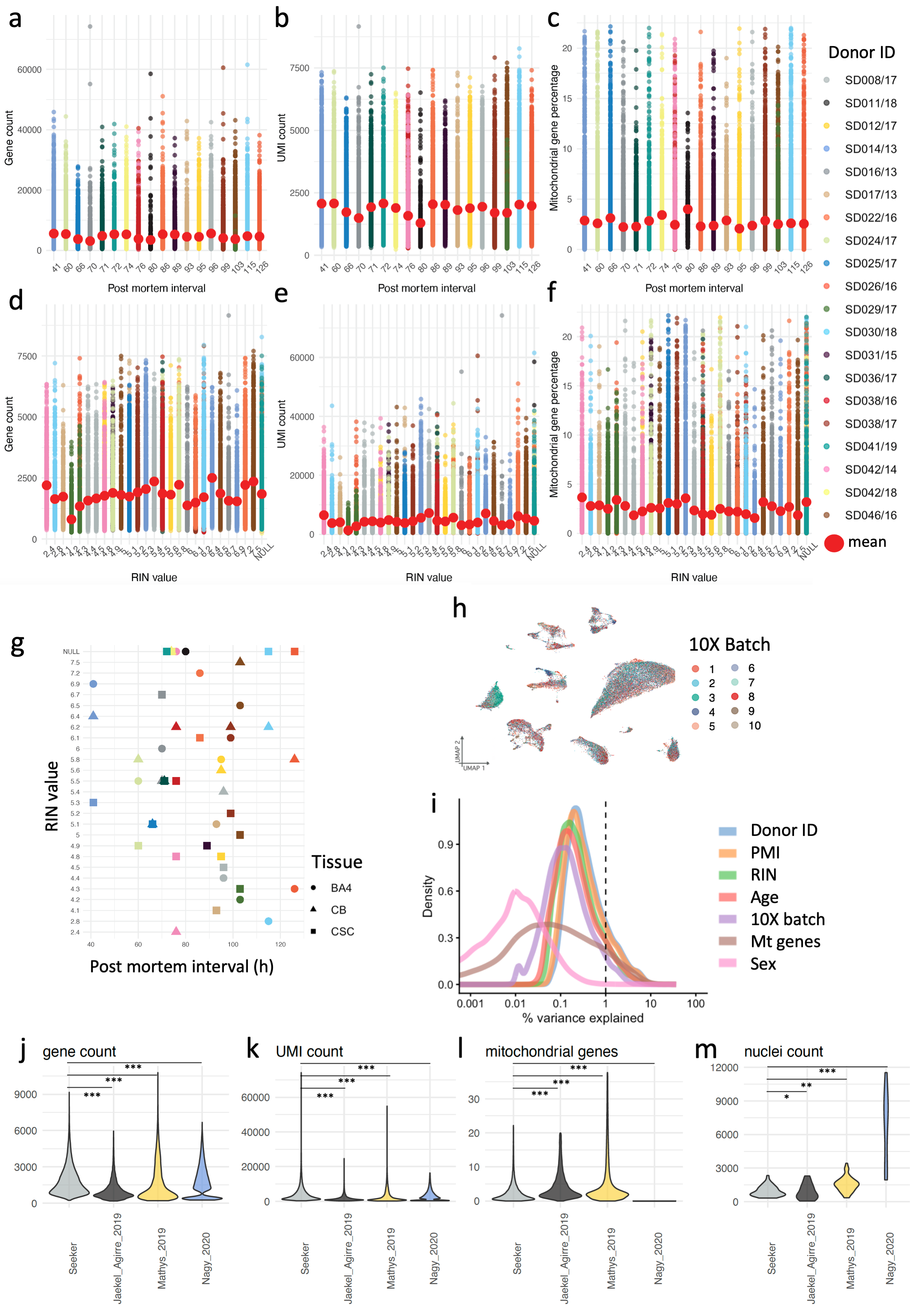


Fig. S2 Quality control measures. Measures of post mortem interval (PMI) with (a) gene count, (b) unique molecular identifier (UMI) count, (c) mitochondrial gene percentage and of RNA integrity number (RIN) with (d) gene count, (e) UMI count and (f) mitochondrial gene percentage show that these potential predictors of sample quality do not predict nucleus quality after filtering. (g) PMI and RIN also do not correlate. (h) UMAP dimensionally reduced representation of the complete dataset shows no strong 10X chip batch effect which is supported by (i) which shows 10X chip batch contributes less to the overall observed variance than other sources of variation such as the individual donor. Comparison of gene (j), UMI (k) mitochondrial gene percentage (i) and nuclei count per sample (m) with previously published snRNAseq datasets of the human CNS tested with linear models and subsequent ANOVAs (* p < 0.05, ** p < 0.01, *** p < 0.001). Note that Nagy et al. removed mitochondrial genes from their published dataset.


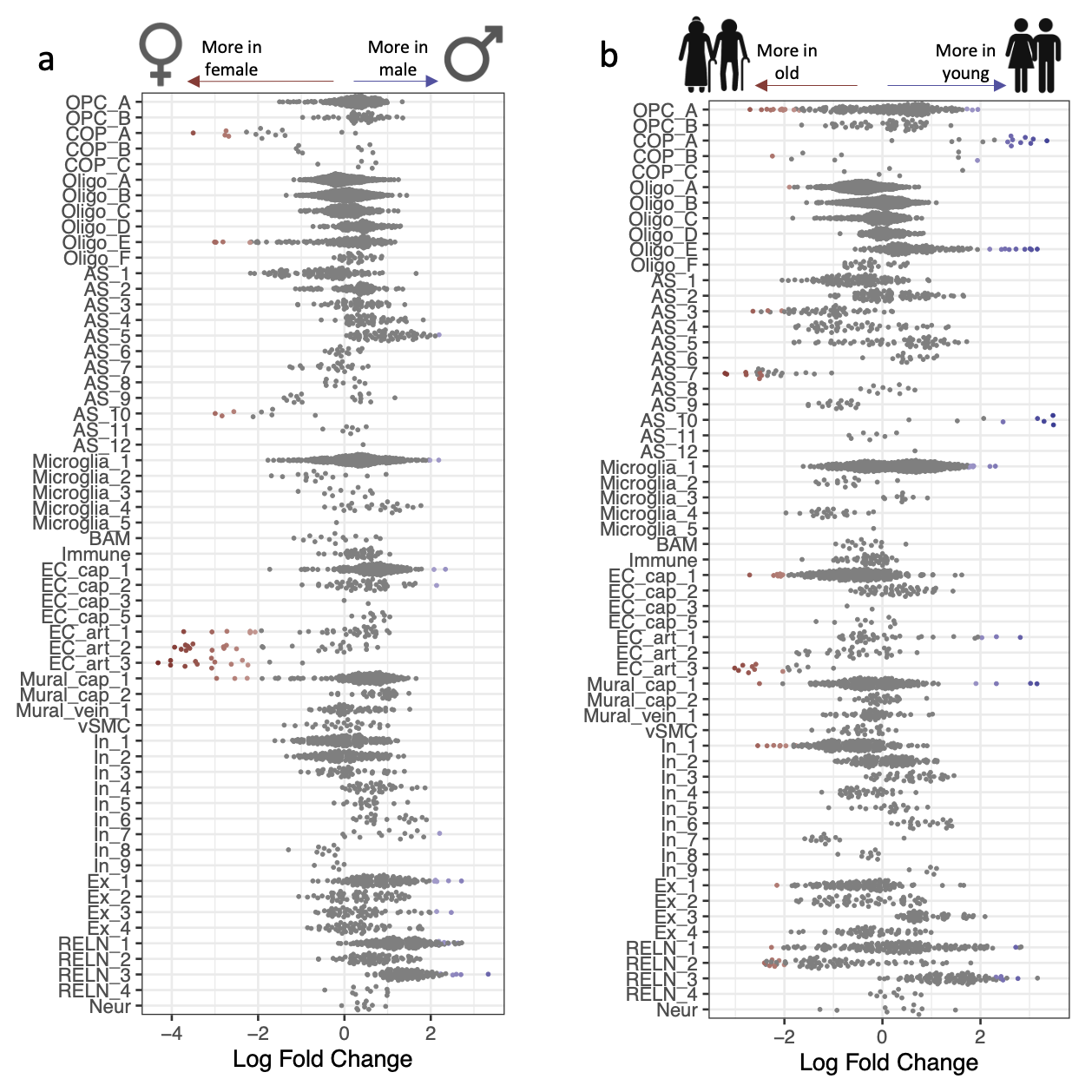


Fig. S 3 Differential abundance across all clusters. Milo was used to test for variation with sex while accounting for donor age and tissue region (a). Milo was also used to test for variation with age while accounting for donor sex and tissue region (b).


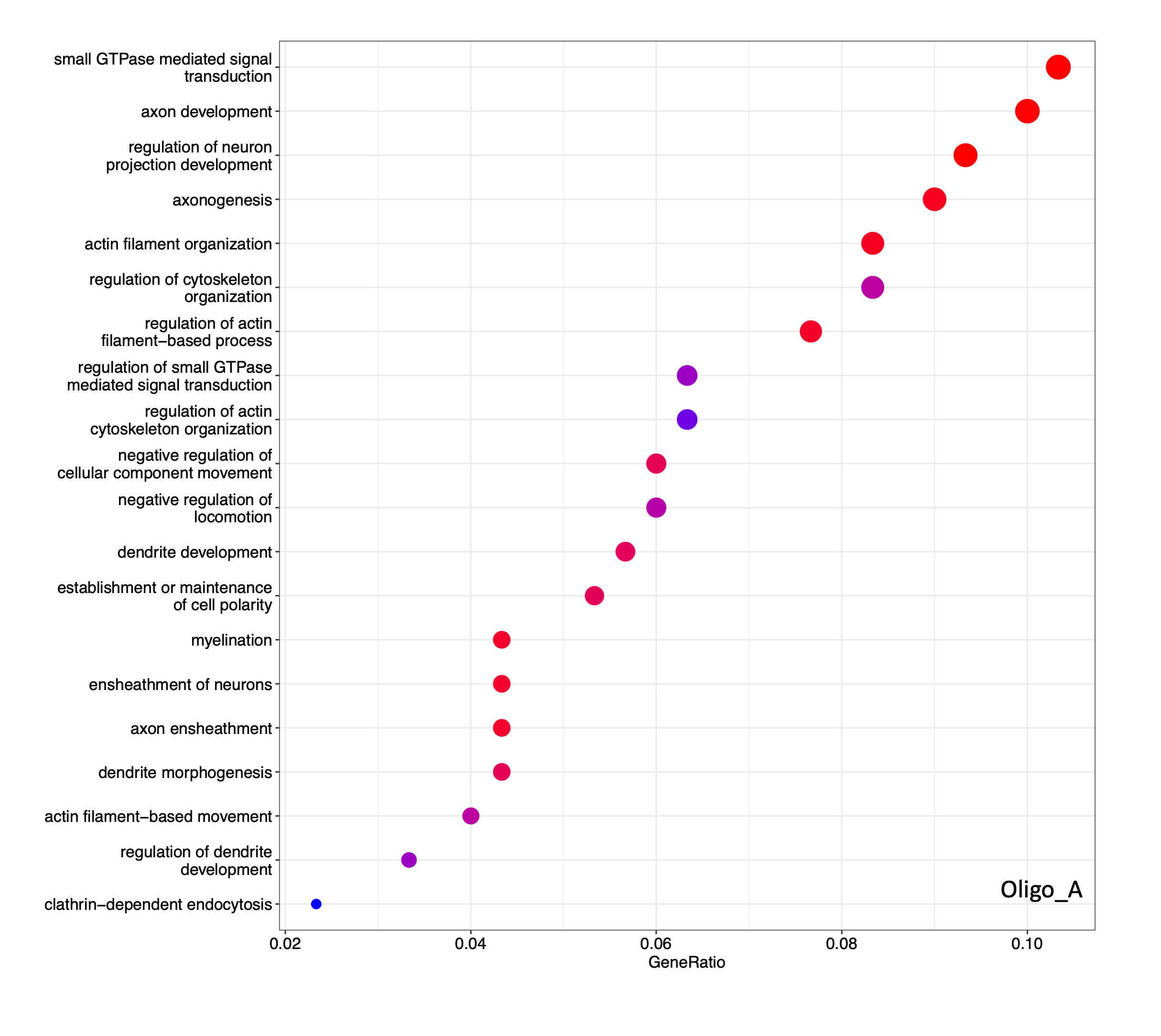


**Fig. S4** Top 20 gene ontology terms associated with cluster Oligo_A.


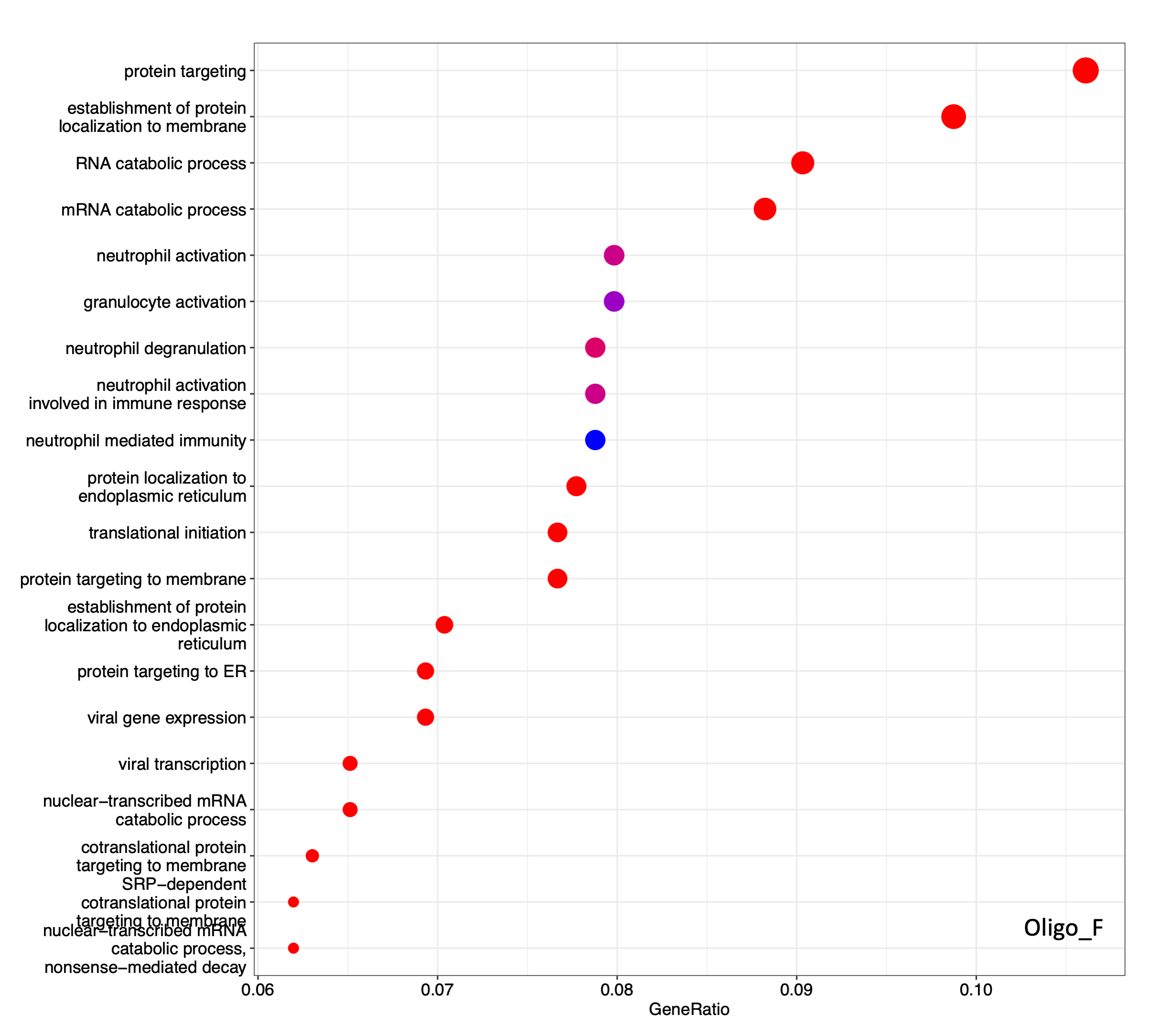


**Fig. S5** Top 20 gene ontology terms associated with cluster Oligo_F.

**
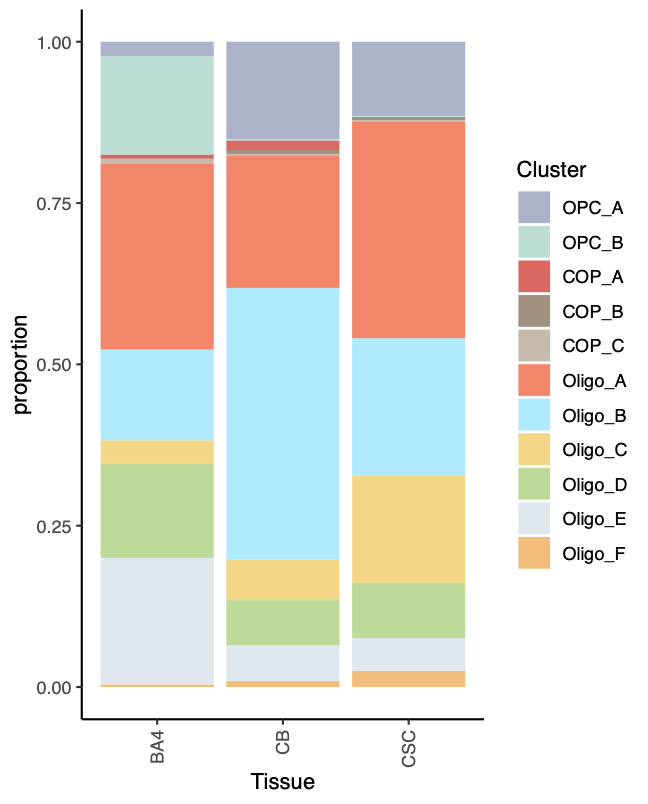
**

Fig. S6 Proportions of oligodendroglia clusters within tissue regions.


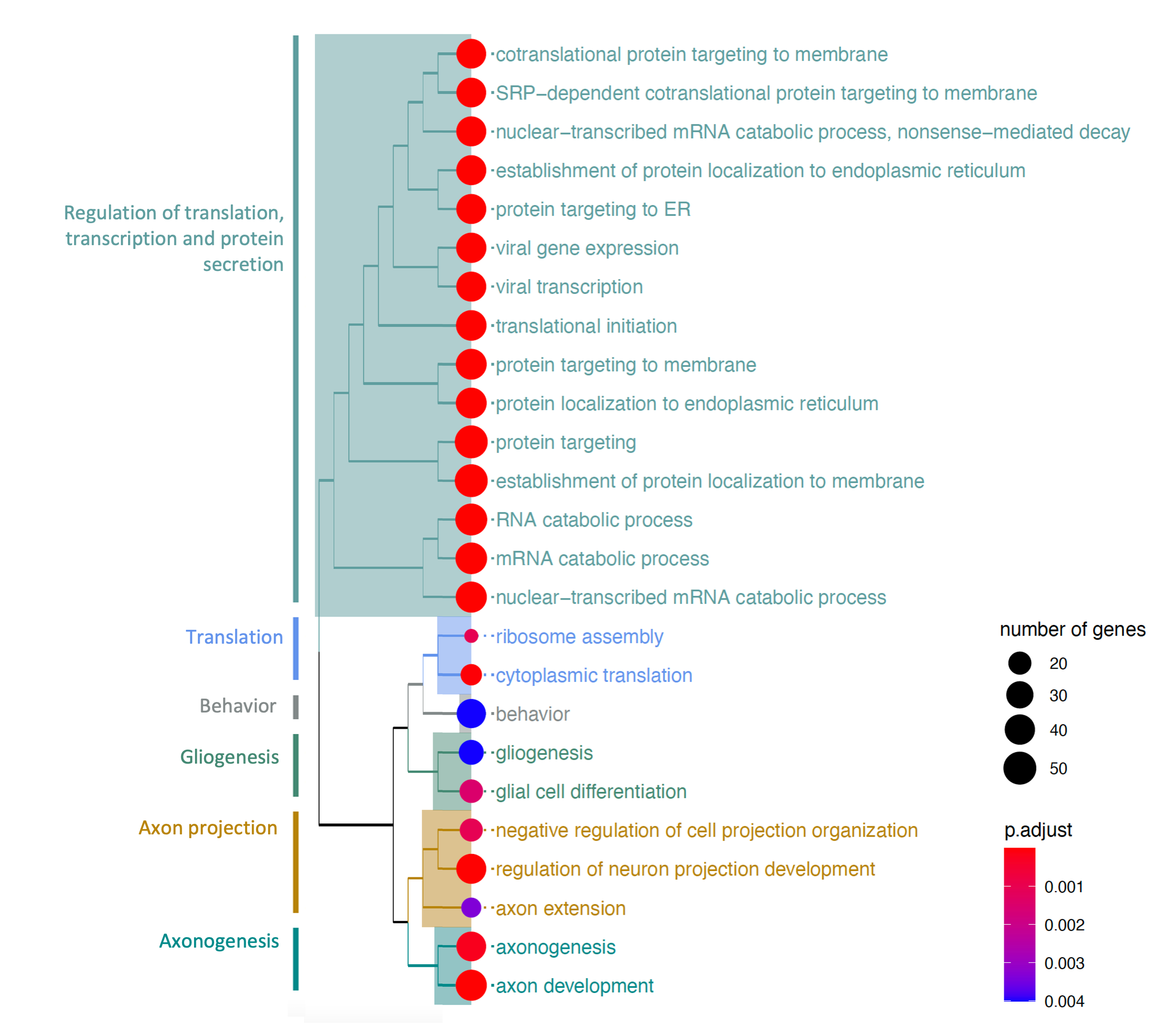


Fig. S7 Gene Ontology of CSC OPCs compared to BA4 OPCs (complete version Fig.3 i).


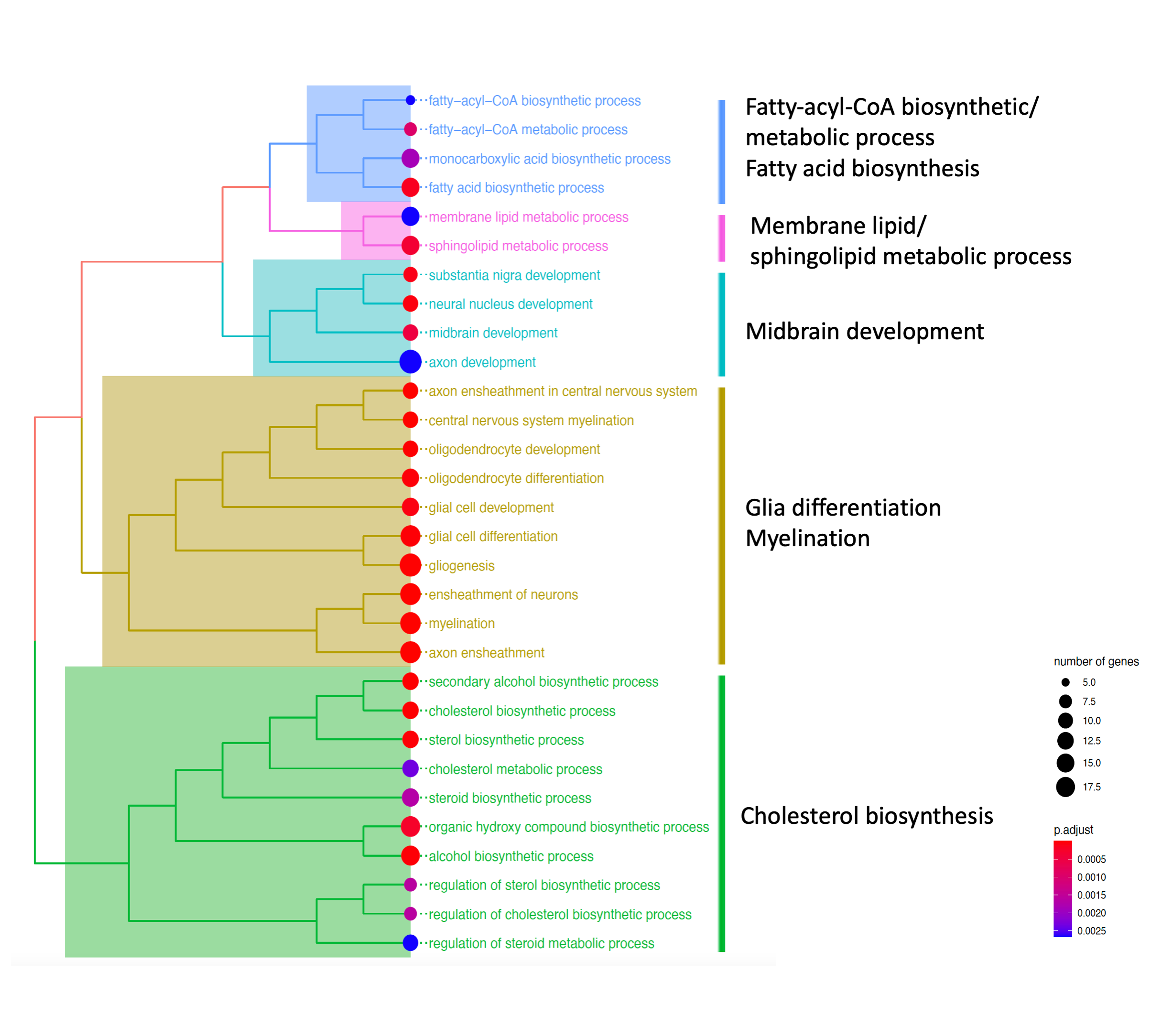


Fig. S8 Gene Ontology analysis of enriched genes in spinal cord oligodendrocytes (complete version to Fig. 3 m).


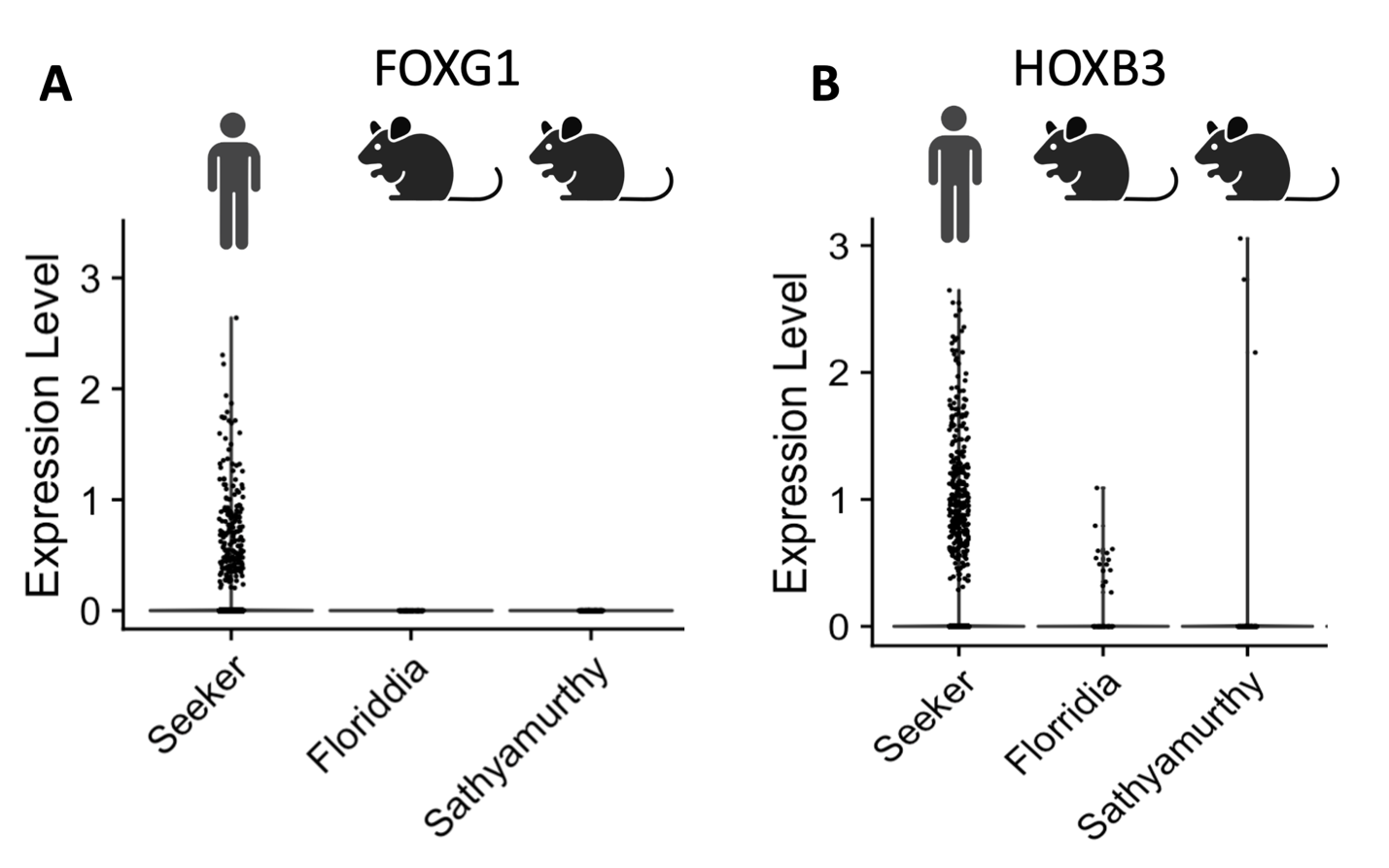


**a**

**b**

Fig. S9 Developmental origin marker expression in adult human and mouse (2, 52) brain and spinal cord data. Example marker for brain (a) and spinal cord (b) marker.


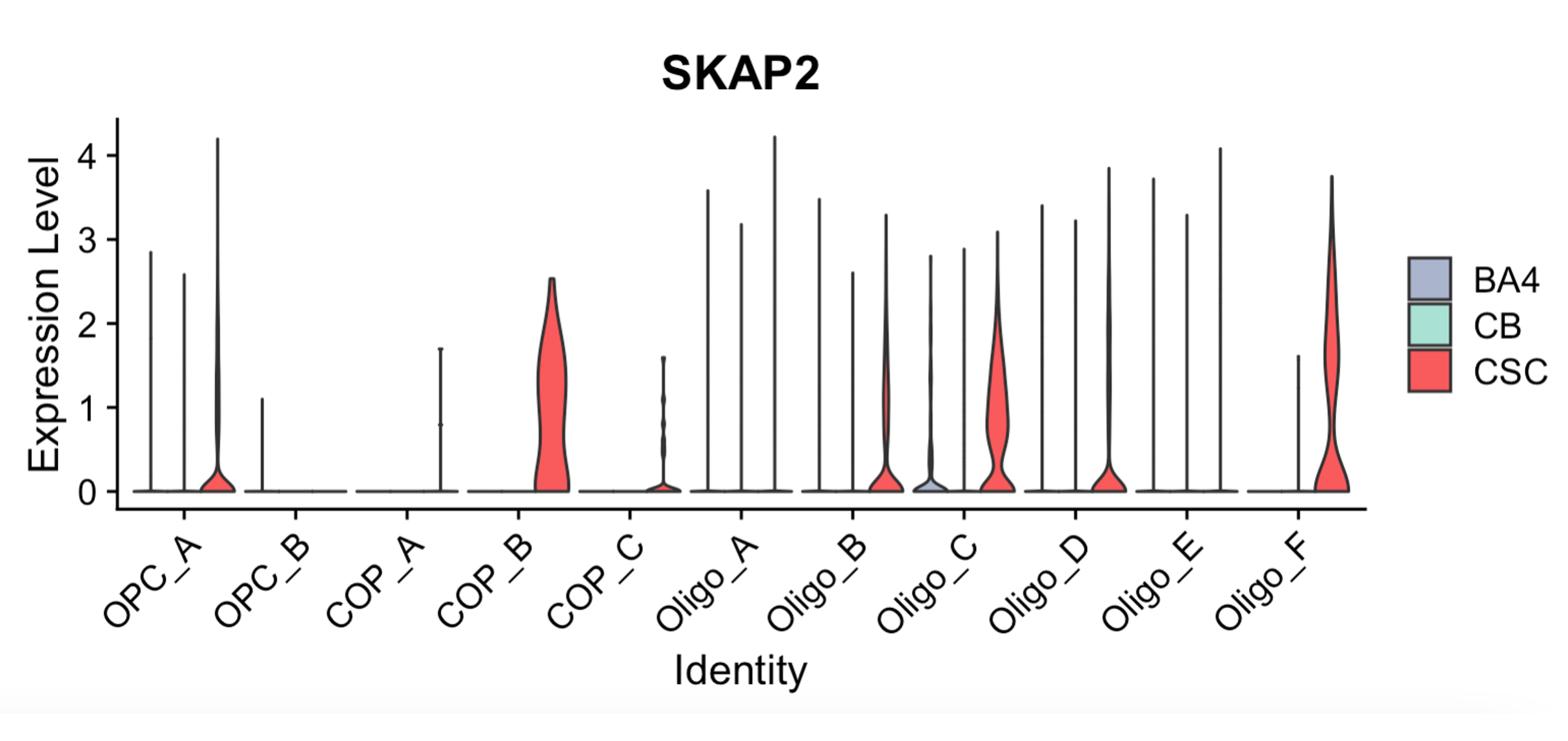


**Fig. S10** SKAP2 expression across oligodendroglia clusters divided by tissue region.


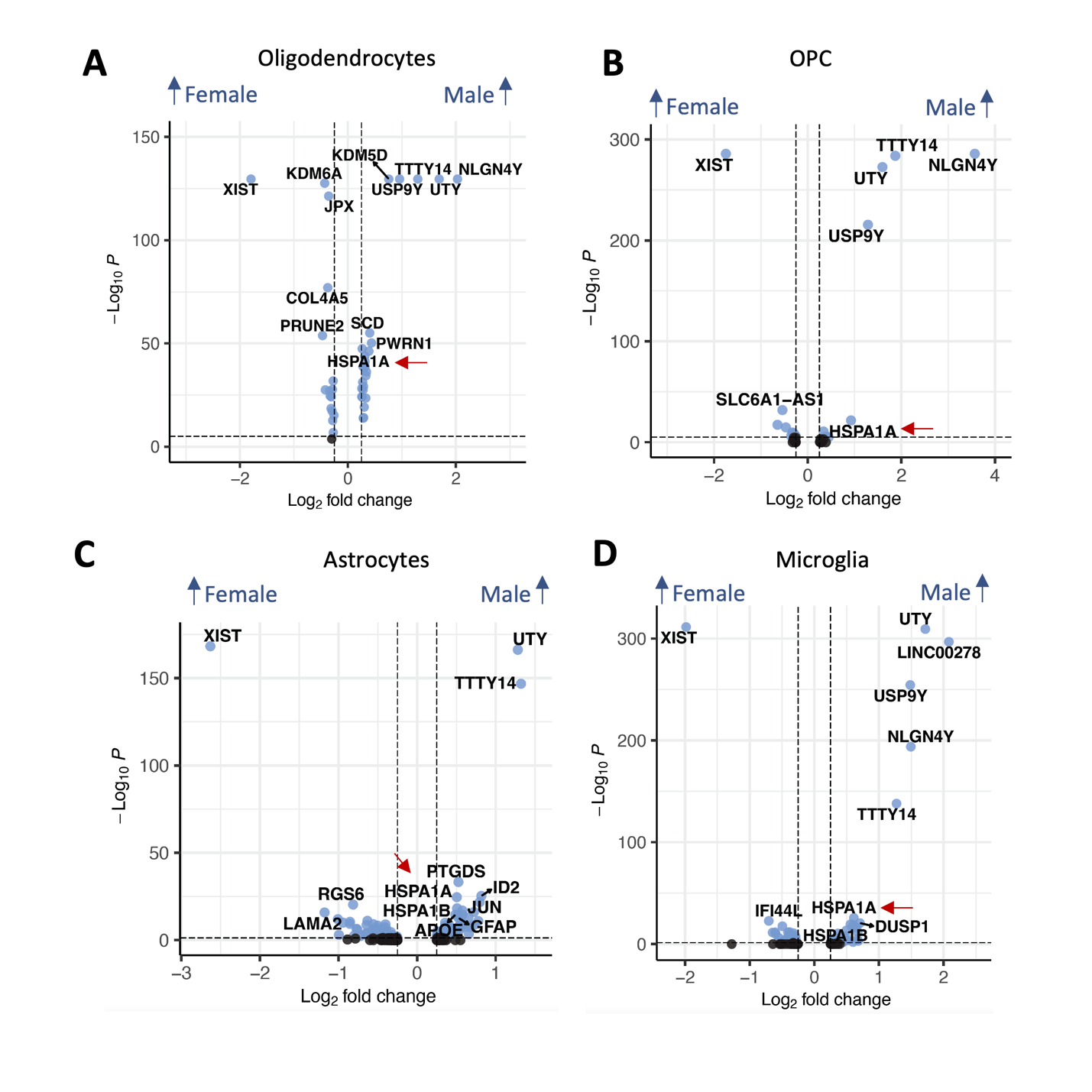


**a**

**b**

**c**

**d**

**Fig. S11** Differentially expressed genes with sex in glia including gonosomal genes for **(a)** oligodendrocytes, **(b)** OPCs, **(c)** Astrocytes and **(d)** Microglia.


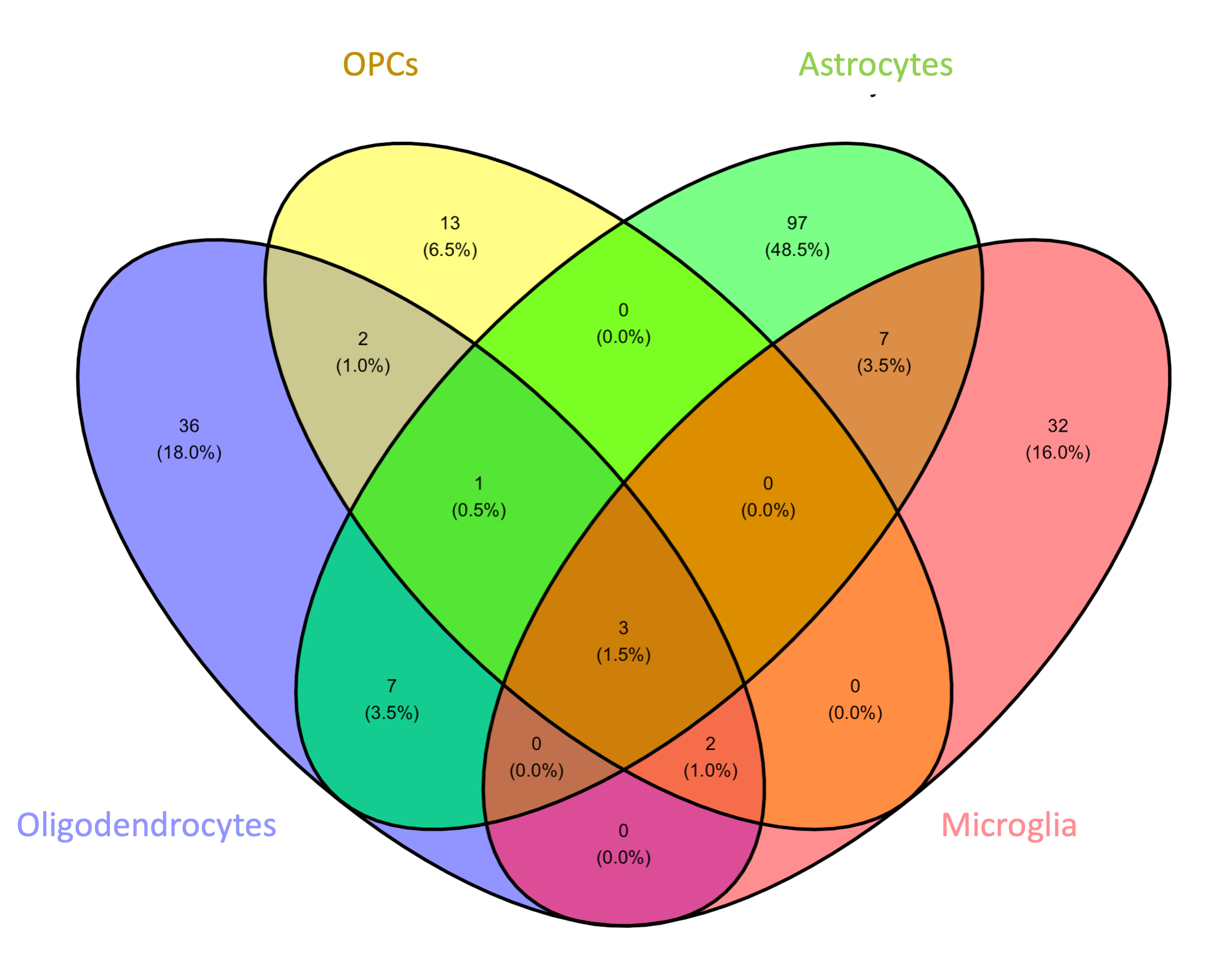


**Fig. S12** Most genes upregulated (log2FC **≥** 0.25, adjusted p-value < 0.05) in male sex compared to female sex are different for each glial type with the exception of the three intersect genes *TTTY14*, *UTY* and *HSP1A1* which are upregulated in all male glia.


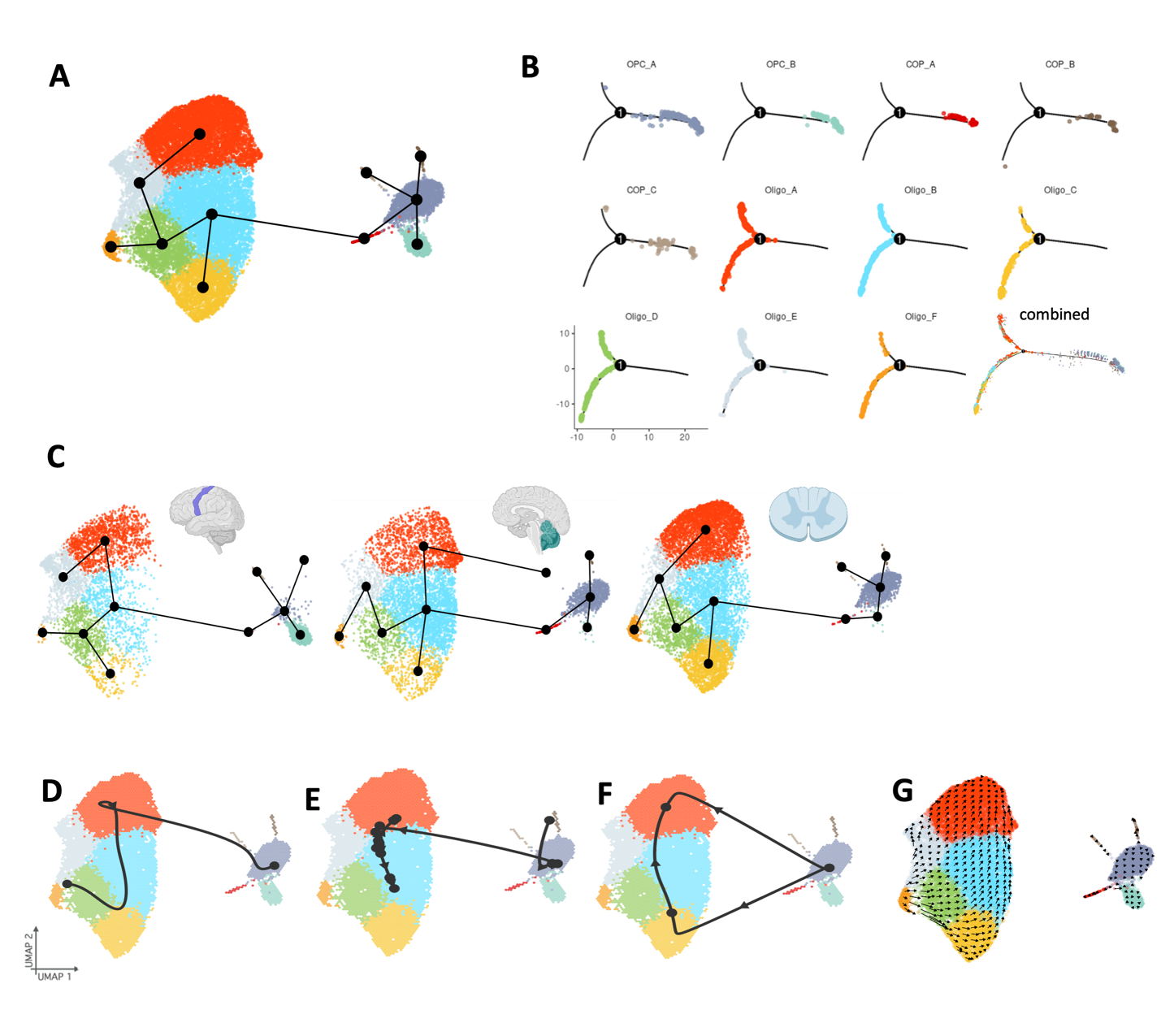


**a**

**b**

**c**

**d**

**e**

**f**

**g**

**Fig. S13** Trajectory inference methods such as Slingshot **(a)** & **(c)**, Monocle **(b)**, Scorpius **(d)**, PAGA-tree **(e)**, Angle **(f)** and scVelo **(g)** do not agree on a likely trajectory. All trajectory inference methods are shown for the complete dataset and Slingshot also individually by CNS tissue **(c)**.


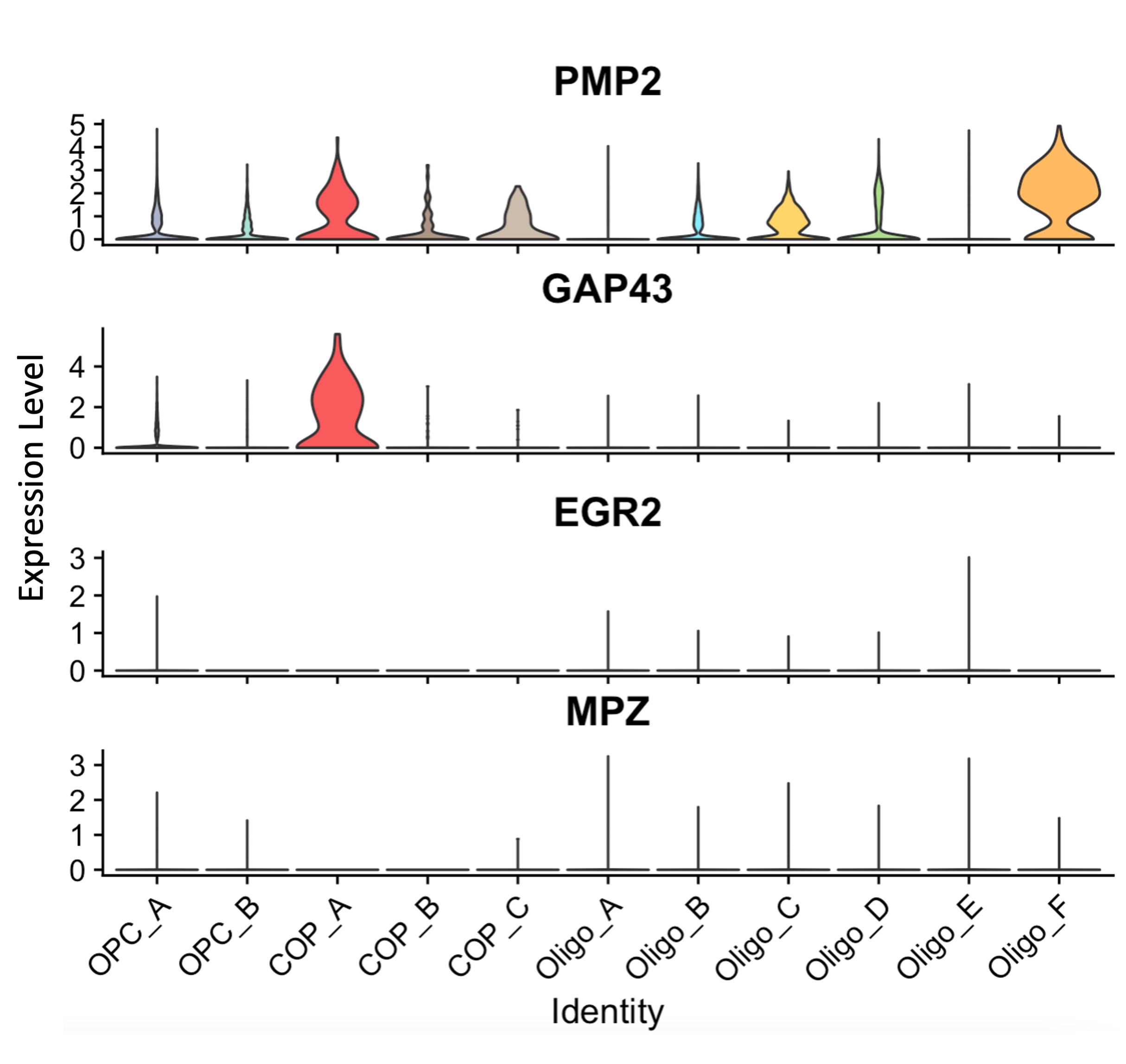


**Fig. S14** Oligo_F expresses PMP2 but no other Schwann cell markers.


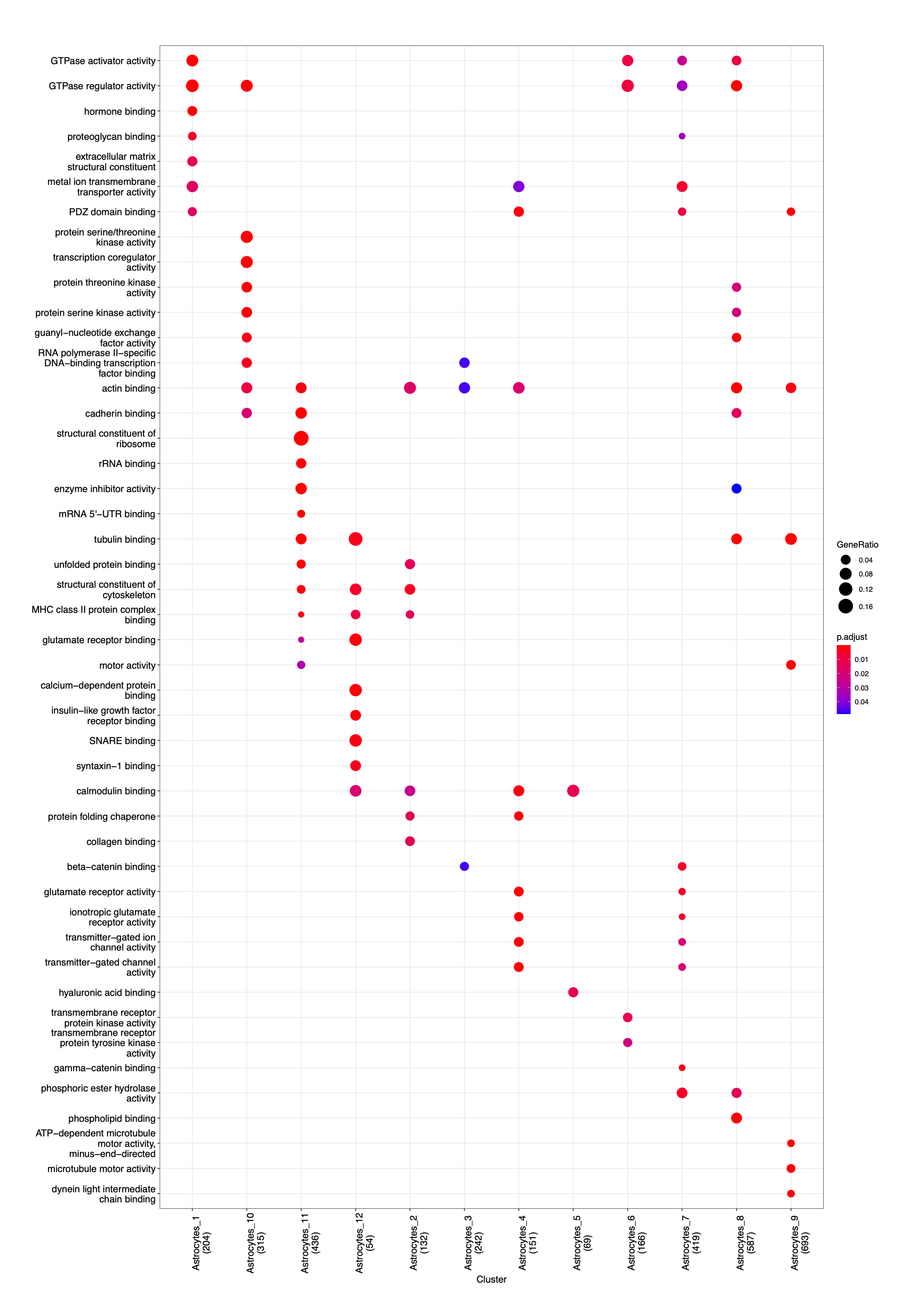


**Fig. S15** Astrocyte Gene ontology with cluster.


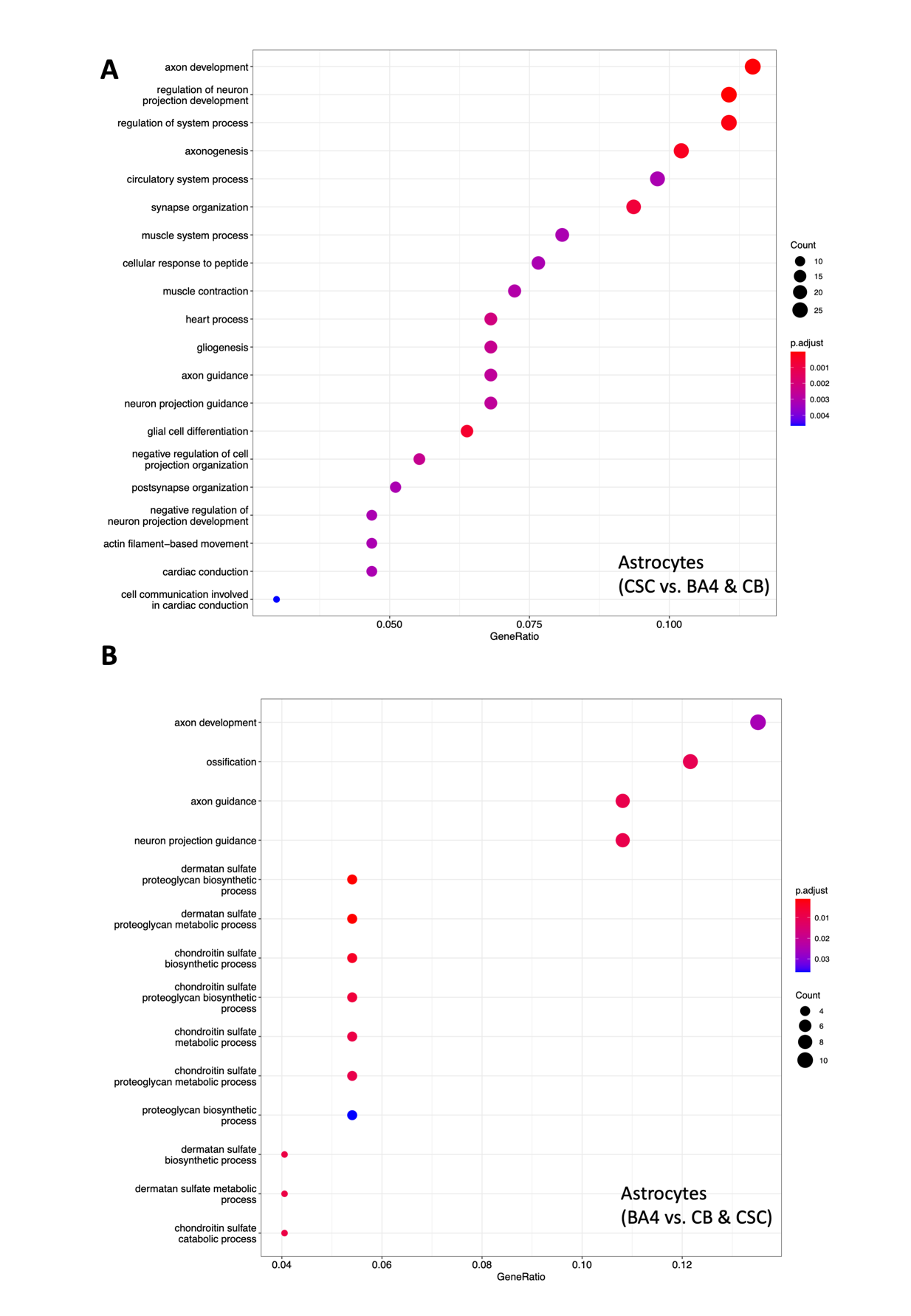


**a**

**b**

**Fig. S16** Top 20 gene ontology terms associated with astrocytes in different CNS regions for **(a)** CSC vs. BA4 & CB and **(b)** BA4 vs. CB & CSC.


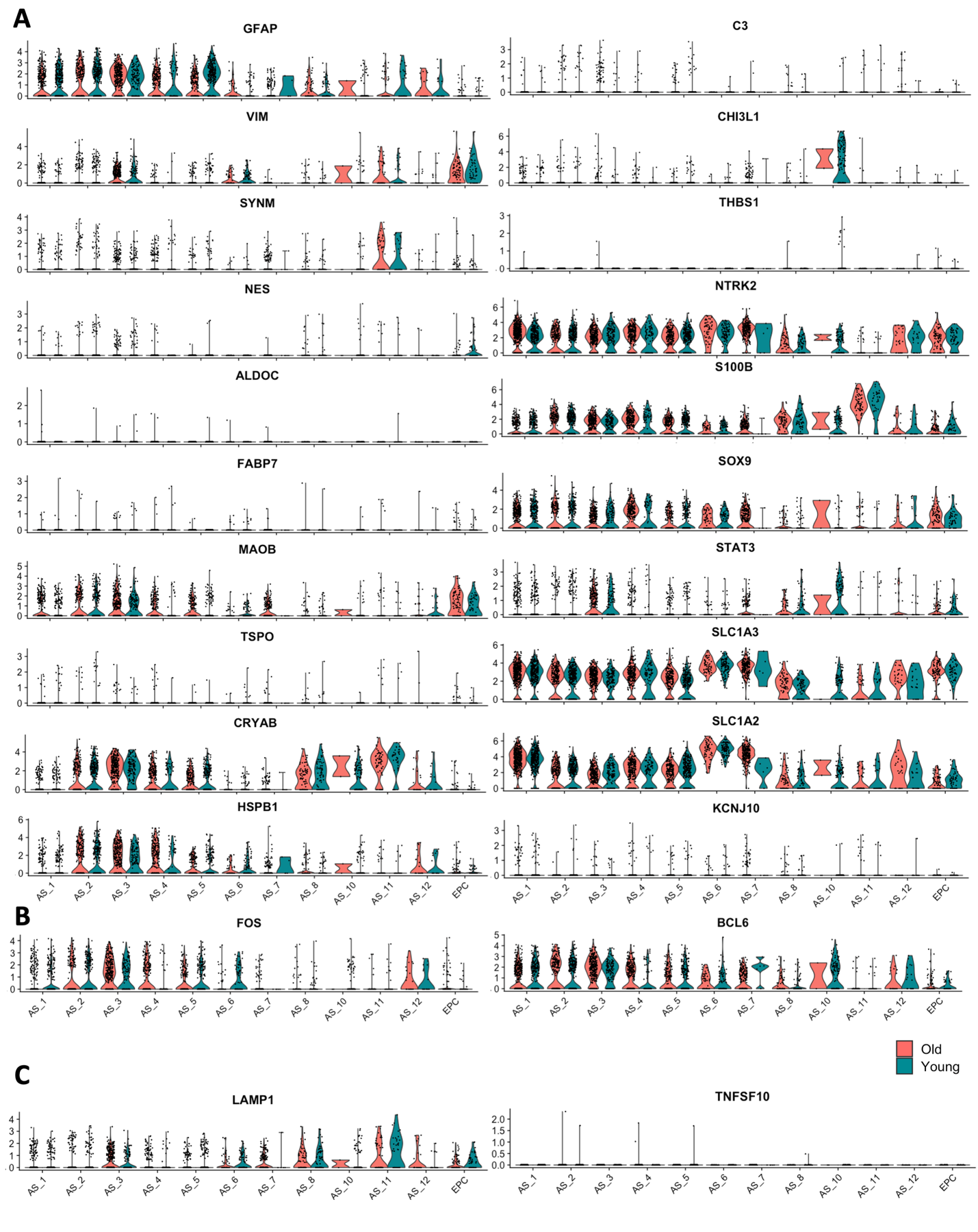


**a**

**b**

**c**

Fig. S17 Activation state markers for astrocytes. a) Genes that are associated with a more activated state *(*90*)*. b) Markers that were upregulated at the borders of multiple sclerosis white matter lesions (24) and c) genes that limit inflammation in the CNS and indicate a more inactivated state *(*91*)*.


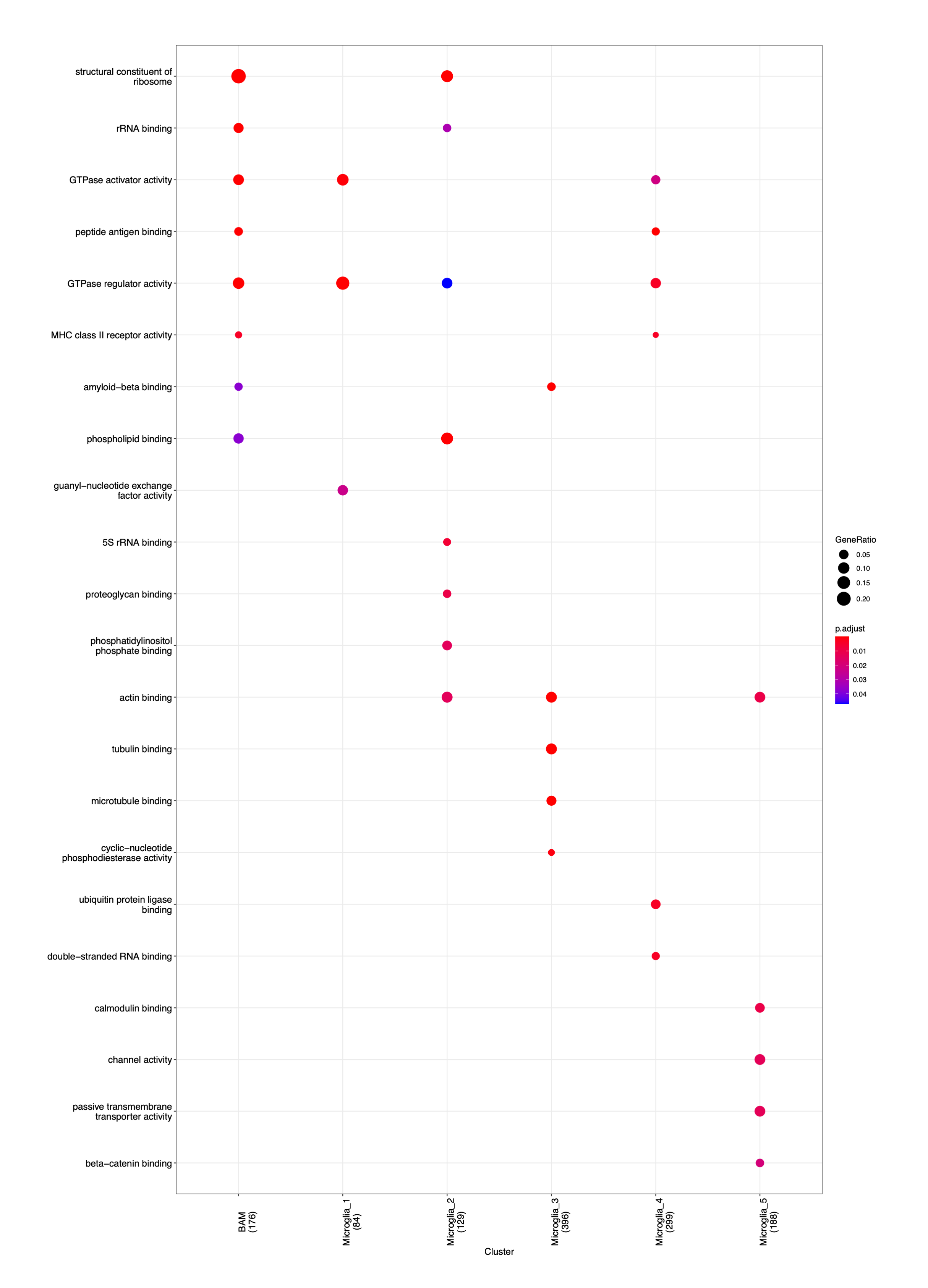
Fig. S18 Microglia gene ontology with cluster.


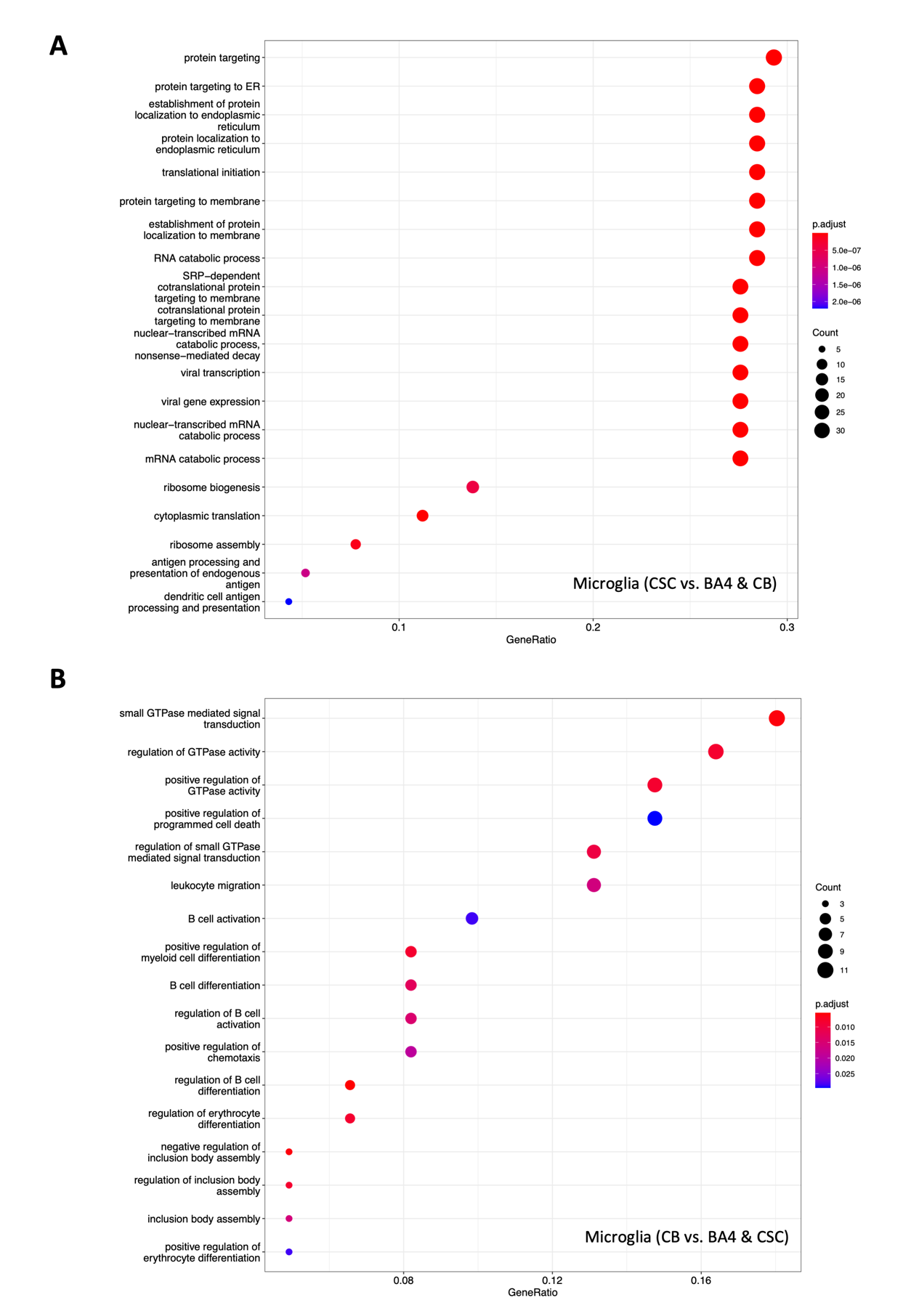


**a**

**b**

Fig. S19 Top 20 gene ontology terms associated with microglia in different CNS regions for (a) CSC vs. BA4 & CB and (b) BA4 vs. CB & CSC.


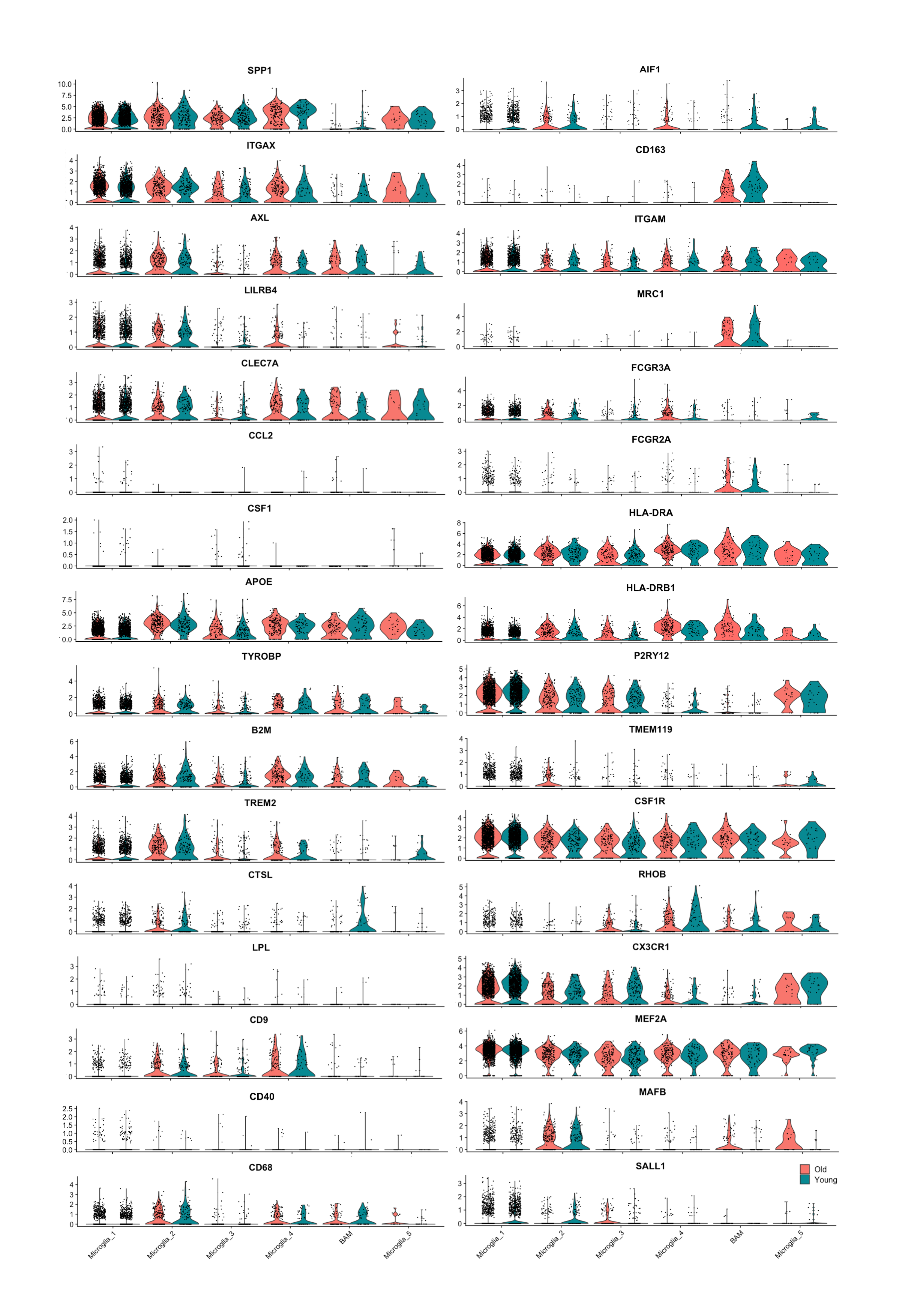


Fig. S20 Microglia gene expression of markers associated with microglia activation and homeostasis.

**Table S1** Donor information for snRNAseq experiment. PMI: post mortem interval in hours.

| individual ID | Sex | Age | Age Group | PMI | Cause of death | Tissue used (after QC) |
| --- | --- | --- | --- | --- | --- | --- |
| SD026/16 | F | 37 | young | 126 | Myocardial fibrosis | BA4, CB, CSC |
| SD031/15 | F | 40 | young | 89 | Hemopericardium | CSC |
| SD041/19 | F | 40 | young | 72 | Unascertained | CSC |
| SD016/13 | F | 44 | young | 70 | Unascertained | BA4, CB, CSC |
| SD017/13 | F | 45 | young | 93 | Coronary artery atherosclerosis | BA4, CSC |
| SD038/17 | M | 34 | young | 99 | Ischaemic heart disease | BA4, CB, CSC |
| SD022/16 | M | 39 | young | 86 | Ischaemic heart disease | BA4, CSC |
| SD038/16 | M | 39 | young | 76 | Suspension by ligature | CB, CSC |
| SD029/17 | M | 40 | young | 103 | Myocardial infarction | BA4, CSC |
| SD046/16 | M | 42 | young | 103 | Suspension by ligature | BA4, CB, CSC |
| SD011/18 | F | 61 | old | 80 | Multi-organ failure secondary to sepsis | BA4 |
| SD008/17 | F | 71 | old | 96 | Ischaemic and hypertensive heart disease | BA4, CB, CSC |
| SD012/17 | F | 71 | old | 95 | Plastic bag suffocation | BA4, CB, CSC |
| SD042/18 | F | 73 | old | 74 | Hypertensive heart disease | BA4, CB, CSC |
| SD014/13 | F | 74 | old | 41 | Pulmonary thromboembolism | BA4, CB, CSC |
| SD030/18 | M | 63 | old | 115 | Ruptured atherosclerotic abdominal aortic aneurysm | BA4, CB, CSC |
| SD042/14 | M | 63 | old | 76 | Complications of metastatic renal cell carcinoma | BA4, CB, CSC |
| SD036/17 | M | 71 | old | 71 | Hemopericardium | CB, CSC |
| SD024/17 | M | 72 | old | 60 | Myocardial infarction | BA4, CB, CSC |
| SD025/17 | M | 73 | old | 66 | Ischaemic heart disease with cardiac enlargement | CB, CSC |

**Table S2** Donor information for validation experiments. PMI: post mortem interval in hours; IF: Immuno-fluorescence; BA4: Brodmann Area 4; CB: Cerebellum; CSC: Cervical spinal cord.

| DONOR INFORMATION | | | | | | | VALIDATION EXPERIMENT | | | | | | | | |
| --- | --- | --- | --- | --- | --- | --- | --- | --- | --- | --- | --- | --- | --- | --- | --- |
| Individual ID | Sex | Age (years) | Age Group | PMI | Cause of death | Tissue Region | PAX3 & PDGFRA (Basescope duplex) | NELL1& PDGFRA (Basescope duplex) | EBF1 & PDGFRA (RNAscope) | SPARC & RBFOX1 & OLIG2 (IF) | HCN2 & SPARC & OLIG2 (IF) | GPNMB & IBA1 & P16P21 (IF) | SPARC & OPALIN & OLIG2 (IF) | OPALIN & RBFOX1 & OLIG2 (IF) | FMN1 & RBFOX1 & OLIG2 (IF) |
| SD004/12 | M | 39 | young | 61 | Suspension by ligature | CB | x |  | x | x |  |  |  |  |  |
|  |  |  |  |  |  | CSC |  | x |  |  |  |  |  |  |  |
| SD008/18 | F | 69 | old | 94 | Ischaemic heart disease | BA4 | x |  |  | x | x | x | x |  |  |
|  |  |  |  |  |  | CSC |  |  |  | x |  |  |  |  |  |
| SD012/15 | M | 45 | young | 51 | Coronary artery thrombosis and MDMA (ecstasy) toxicity | BA4 | x | x |  | x | x |  | x | x |  |
|  |  |  |  |  |  | CSC | x |  | x | x |  |  |  |  |  |
| SD022/13 | M | 45 | young | 74 | Coronary artery thrombosis | CB |  |  | x |  |  |  |  |  |  |
|  |  |  |  |  |  | CSC | x |  |  | x |  |  |  |  |  |
| SD026/16 | F | 37 | young | 126 | Myocardial fibrosis - cause uncertain | CSC | x |  | x | x |  |  |  |  |  |
| SD030/12 | F | 71 | old | 41 | Ischaemic and hypertensive heart disease | CB |  |  |  |  |  |  |  |  |  |
|  |  |  |  |  |  | CSC | x |  | x | x |  | x |  |  |  |
| SD031/14 | F | 45 | young | 40 | Suspension by ligature | BA4 | x |  |  | x | x |  | x |  | x |
|  |  |  |  |  |  | CB | x |  | x |  |  |  |  |  |  |
| SD035/15 | M | 69 | old | 90 | Ischaemic heart disease | CB | x |  |  |  |  |  |  |  |  |
|  |  |  |  |  |  | CSC |  |  | x |  |  |  |  |  |  |
| SD038/15 | M | 44 | young | 95 | Ischaemic heart disease | BA4 | x |  |  | x |  |  |  |  |  |
| SD039/14 | M | 60 | old | 65 | Coronary artery thrombosis | CB |  |  |  |  |  | x |  |  |  |
|  |  |  |  |  |  | CSC | x |  | x | x |  |  | x |  |  |
| SD046/17 | F | 65 | old | 76 | Ischaemic and hypertensive heart disease | CB | x |  | x | x |  |  |  |  |  |
| SD015/12 | M | 70 | old | 74 | Hypertensive and ischaemic heart disease | CB |  |  | x |  |  | x |  |  |  |
|  |  |  |  |  |  | CSC | x |  | x |  |  | x |  | x |  |
| SD024/14 | M | 38 | young | 36 | Ischaemic heart disease | CSC | x |  |  |  |  |  |  |  |  |
| SD011/18 | F | 61 | old | 80 | Multi-organ failure secondary to sepsis | CB | x |  | x | x |  |  |  |  |  |
|  |  |  |  |  |  | CSC |  |  |  |  | x |  |  | x |  |
| SD021/17 | M | 67 | old | 68 | Ischaemic heart disease | BA4 | x | x |  | x |  | x |  | x | x |
| SD001/07 | F | 37 | young | 46 | Bronchopneumonia | CSC |  | x |  |  |  |  | x |  |  |
| SD037/14 | F | 41 | young | 50 | Unascertained | CSC |  | x | x |  |  |  |  |  |  |
| SD042/18 | F | 73 | old | 74 | Hypertensive heart disease | BA4 |  | x |  | x |  | x |  | x | x |
|  |  |  |  |  |  | CSC |  |  | x |  | x | x |  | x |  |
| SD034/15 | M | 69 | old | 49 | Ischaemic and hypertensive heart disease | CB |  |  |  | x |  |  |  |  |  |
| SD061/13 | F | 40 | young | 77 | Bronchial asthma | CSC |  |  | x |  |  |  | x |  |  |
| SD029/11 | F | 42 | young | 86 | Bronchial asthma | CB |  |  | x |  |  |  |  |  |  |
| SD006/14 | M | 60 | old | 52 | Bronchopneumonia | CB |  |  | x |  |  |  |  |  |  |
| SD008/14 | M | 43 | young | 96 | Coronary artery thrombosis | CSC |  |  | x |  |  |  |  |  | x |
